# Immune infiltration, aggressive pathology, and poor survival outcomes in RECQL helicase deficient breast cancers

**DOI:** 10.1016/j.neo.2023.100957

**Published:** 2023-12-21

**Authors:** Ayat Lashen, Abdulbaqi Al-Kawaz, Jennie N Jeyapalan, Shatha Alqahtani, Ahmed Shoqafi, Mashael Algethami, Michael Toss, Andrew R Green, Nigel P Mongan, Sudha Sharma, Mohammad R Akbari, Emad A Rakha, Srinivasan Madhusudan

**Affiliations:** a Nottingham Breast Cancer Research Centre, Nottingham Biodiscovery Institute, School of Medicine, University of Nottingham, University Park, Nottingham NG7 3RD, UK; b Department of Pathology, Nottingham University Hospital, City Campus, Hucknall Road, Nottingham NG51PB, UK.; c Department of Pharmacology, Weill Cornell Medicine, New York, NY 10065, USA; d Department of Biochemistry and Molecular Biology, College of Medicine, Howard University, 520 W Street, NW, Washington, DC 20059, USA; e Women's College Research Institute, Women's College Hospital, University of Toronto, Toronto, Canada; f Institute of Medical Science, Faculty of Medicine, University of Toronto. Toronto, Canada; g Dalla Lana School of Public Health, University of Toronto, Toronto, Canada; h Department of Oncology, Nottingham University Hospitals, Nottingham NG51PB, UK

**Keywords:** RECQL, CD8, FOXP3, IL17, PDL1, PD1, Immune cell infiltration, Breast cancer, DCIS, Local recurrence

## Abstract

RECQL is essential for genomic stability. Here, we evaluated RECQL in 449 pure ductal carcinomas *in situ* (DCIS), 152 DCIS components of mixed DCIS/invasive breast cancer (IBC) tumors, 157 IBC components of mixed DCIS/IBC and 50 normal epithelial terminal ductal lobular units (TDLUs). In 726 IBCs, CD8+, FOXP3+, IL17+, PDL1+, PD1+ T-cell infiltration (TILs) were investigated in RECQL deficient and proficient cancers. Tumor mutation burden (TMB) was evaluated in five *RECQL* germ-line mutation carriers with IBC by genome sequencing. Compared with normal epithelial cells, a striking reduction in nuclear RECQL in DCIS was evident with aggressive pathology and poor survival. In RECQL deficient IBCs, CD8+, FOXP3+, IL17+ or PDL1+ TILs were linked with aggressive pathology and shorter survival. In germline *RECQL* mutation carriers, increased TMB was observed in 4/5 tumors. We conclude that RECQL loss is an early event in breast cancer and promote immune cell infiltration.

## Introduction

DNA helicases are molecular motors that unwind DNA and essential for the maintenance of genomic stability [1]. RECQL (also known as RECQ1 or RECQL1) belongs to the RecQ family of DNA helicases [2]. RECQL 3´-5’ helicase activity is required to unwind DNA, an essential step required during DNA replication and DNA repair. RECQL is implicated in homologous recombination. It interacts with PARP1, RPA, RAD51, Top3α, EXO1, MSH2/6, MLH1-PMS2 and Ku70/80 during DNA repair [3,4]. Preclinically, RECQL depletion leads to increased spontaneous sister chromatid exchanges, chromosomal instability, and DNA damage accumulation in cells [3,4]. Emerging data indicates a role for RECQL in breast cancer pathogenesis. We have previously shown that germ-line mutations in *RECQL* are extremely rare and may increase the risk of developing breast cancer [5]. In sporadic invasive breast cancers (IBC), we demonstrated that RECQL deficiency at the transcriptomic and proteomic levels are associated with aggressive breast cancer phenotypes and poor patient survival [6]. More recently, we validated these observations in an independent clinical cohort of ER-positive breast cancer where RECQL deficiency was associated with poor survival [7]. Pre-clinically, in ER-positive breast cancer cells, we observed that RECQL interacts with the FOXA1 transcription factor and regulates expression of the *ESR1* gene which encodes the ERα protein [8]. These studies suggest a role for RECQL in breast cancer pathogenesis and prognosis. We thus hypothesized that RECQL deficiency may be an early event during breast cancer pathogenesis. Moreover, RECQL deficient genomically unstable tumors may have increased neoantigens expression that could promote tumoral T-cell infiltration and aggressive pathology.

## Results

**Fig. 1:**
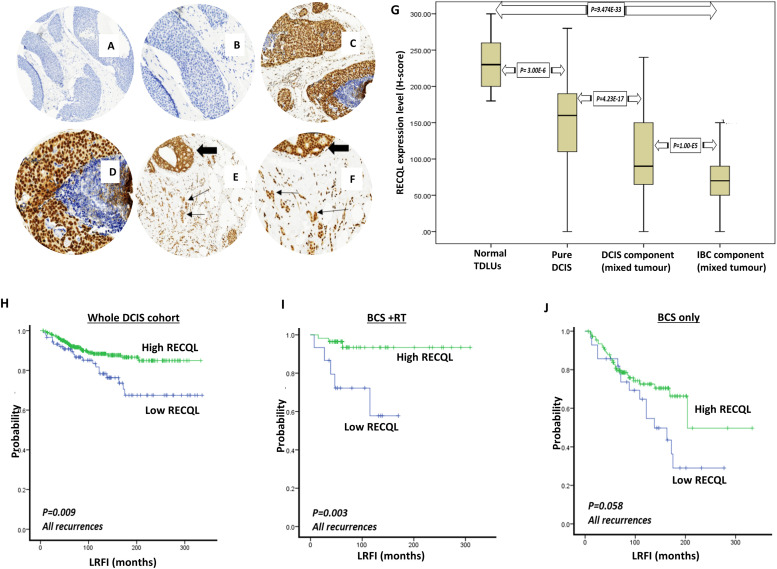
RECQL nuclear protein expression in DCIS. A&B. DCIS negative stain (4X and 10X power magnification respectively). C&D. Intense nuclear staining in pure DCIS (4X and 10X power magnification respectively). E&F. Stronger nuclear staining in DCIS component (thick arrow) than in invasive component (thin arrow) (4X and 10X power magnification respectively). G. RECQL nuclear protein expression boxplot showing higher nuclear RECQL expression present in the normal TDLUs, decreased in pure DCIS series and further decrease in DCIS component and the lowest level was seen in the IBC component of the mixed DCIS/IBC cohort.

RECQL protein expression was assessed, using tissue microarrays (TMA), in 50 normal Terminal ductal lobular units (TDLUs), 449 pure DCIS, 152 DCIS components of DCIS/IBC, and 157 IBC components of DCIS/IBC . Patient demographics are summarized in Supplementary Table 1. IHC revealed strong nuclear expression of RECQL in the normal luminal epithelial cells of the adjacent TDLUs and lower nuclear expression in the cancerous epithelial cells with occasional inflammatory cells. Cytoplasmic expression was not detectable (Fig. 1A–F). Median nuclear Histochemical (H)-scores were 230, 160, 90, and 70 in adjacent normal TDLUs, primary DCIS, the DCIS component of DCIS/IBC, and the IBC component of DCIS/IBC tumors respectively. Assessment of RECQL expression revealed higher RECQL levels in the normal epithelial cells of the adjacent TDLUs than in the cancerous epithelial cells of the pure DCIS (*p =* 3.0 × 10 ^−6^) (Fig. 1G). In the DCIS component of DCIS/IBC tumors, RECQL expression was lower compared to pure DCIS (mean H-scores 90 versus 160) (*p =* 4.2 × 10 ^−17^) (Fig. 1G). In the IBC component of DCIS/IBC tumors, RECQL expression was lower compared to the DCIS component of DCIS/IBC tumors (mean H-scores 70 versus 90) (*p =* 1.0 × 10 ^−5^) (Fig. 1G). Overall, these observations reveal a clear reduction in RECQL level from normal epithelial cells to invasive cancer cells (mean H-scores 230 versus 70) (*p =* 9.4 × 10 ^−44^) (Fig. 1G).

### RECQL and ductal carcinoma *in situ* (DCIS)

In pure DCIS, low RECQL expression was observed in 89/449 (20 %) cases and high expression in 360/449 (80 %) cases. Low RECQL expression showed an association with high DCIS grade (*p =* 0.028) but not with other parameters (Supplementary Table 2). In the DCIS component of DCIS/IBC tumors, low nuclear RECQL expression was associated with large DCIS size (*p =* 0.010) and high DCIS grade (*p =* 0.045) only (Supplementary Table 3). Univariate analysis in the pure DCIS showed that low RECQL expression was associated with shorter local recurrence free interval (LRFI) (both *in situ* and invasive recurrence) (*p =* 0.009) (Fig. 1H). In pure DCIS, Low RECQL expression was also associated with shorter LRFI in patients treated by breast conserving surgery (BCS) followed by adjuvant RT (*p =* 0.003) (Fig. 1I) but not in patients treated with BCS only (*p =* 0.058) (Fig. 1J). Multivariate Cox regression analysis for recurrence free interval in the pure DCIS series demonstrated that low RECQL expression was an independent poor prognostic factor of all recurrences in patients treated with BCS (p=0.028; HR = 0.538; 95 % CI = 0.309 - 0.936). Other independent risk factors included age of the patients at the time of diagnosis (*p =* 0.004; HR = 0.538; 95 % CI = 0.309–0.936) and DCIS tumor size (*p =* 0.001; HR = 0.398; 95 % CI = 0 .228-0.695) (Supplementary Table 4). Furthermore, the multivariate analysis in the pure DCIS series with IBC recurrence also demonstrated that low RECQL expression was an independent prognostic factor for tumor recurrence in patients treated with BCS (*p =* 0.029; HR = 2.913; 95 % CI = 1115–7.608). Other independent risk factors included age of the patients at the time of diagnosis, DCIS tumor size, and DCIS grade (Supplementary Table 4).

Taken together, the data provides the first clinical evidence that RECQL deficiency in DCIS promotes aggressive phenotype and adverse prognostic significance.

**Fig. 2:**
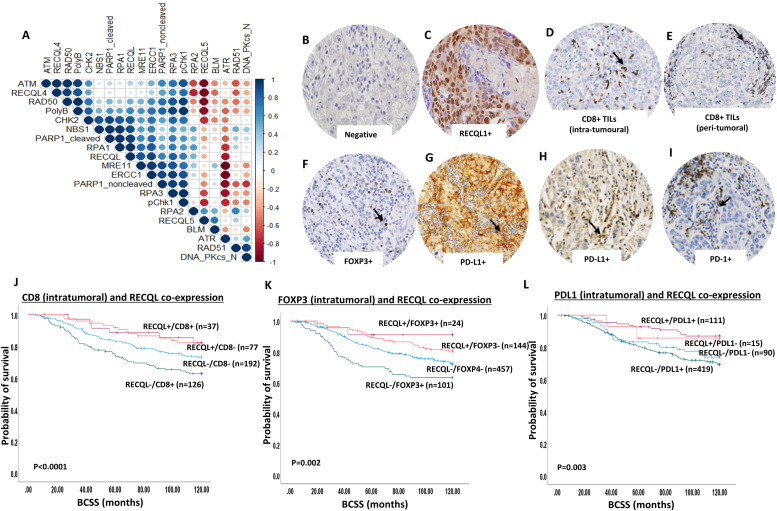
RECQL deficiency and immune infiltration in IBC cohort. A. Correlation matrix showing the correlation between levels of RECQL and various DNA repair markers. B. RECQL negative tumor (image at 20x magnification). C. RECQL positive tumor (image at 20x magnification). D. Invasive carcinoma showing CD8 + intra-tumoural lymphocytic infiltrate (image at 20x magnification). E. Invasive carcinoma showing CD8+ peri-tumoural lymphocytic infiltrate (image at 20x magnification). F. Invasive carcinoma showing FOXP3+ intra-tumoral lymphocytic infiltrate (image at 20x magnification). G. PDL1 expression in tumor cells (image at 20x magnification). H. Invasive carcinoma showing PDL1 positive intra-tumoral lymphocytic infiltrate (image at 20x magnification). I. Invasive carcinoma showing PD1 positive intra-tumoral lymphocytic infiltrate (image at 20x magnification). J. Kaplan Meir curve showing prognostic significance of intra-tumoral CD8+TILs in RECQL deficient or proficient tumors (image at 20x magnification). K. Kaplan Meier curve showing prognostic significance of intra-tumoral FOXP3+TILs in RECQL deficient or proficient tumors. L. Kaplan Meier curve showing prognostic of intra-tumoral PDL1+TILs in RECQL deficient or proficient tumors.

We have previously shown that low levels of RECQL protein is associated with aggressive IBC including larger tumor size, lymph node positivity, high tumor grade, high mitotic index, pleomorphism, dedifferentiation, ER negativity and poor survival [6,7]. We hypothesized that RECQL deficiency induced genomic instability [3,4] will not only lead to increased mutagenicity/carcinogenicity but can also increase neoantigen load on tumor cell surface resulting in increased immunogenicity and T-cell infiltration [9]. To address this possibility, we first correlated RECQL expression to a panel of DNA repair marker expression in IBC cohort (Fig. 2A). T-cell infiltration [CD8+, FOXP3+, and PD1+ cells] and tumor PD-L1 expression (Fig. 2B–I) was then investigated in RECQL deficient and RECQL proficient IBC. CD8+, FOXP3+ and PD1+ T-cells were evaluated within tumor cell nests, adjacent or distant stroma. Patient demographics of the IBC cohort (n=726) are shown in supplementary Table 5. Immunohistochemical staining protocol is shown in Supplementary Table 6 and described previously [10]. A shown in Fig. 2A and Supplementary Table 7, we observed a positive correlation between RECQL and RECQL4, RECQL5, BLM, RPA1, Ku70, MRE11, RAD50, BRCA1, XRCC1, Polymerase beta, pCHK1, CHK2, DNA-PKcs, ERCC1 and PARP1 (all p values <0.0001).

**CD8+ T- cell infiltration in RECQL deficient IBC:** Breast cancers with enhanced immunogenicity will be susceptible to CD8^+^ T cell infiltration. The number of CD8+ T-cells were counted in each tumor core. CD8+ T cells were counted in three locations in each tumor: intra-tumoral compartment (within the tumor cell nests), within the adjacent stroma (defined as CD8+ cells within one tumor cell diameter of the tumor) and within the distant stroma (defined as > one tumor cell diameter away from the tumor). The total number of CD8+ T cells was determined by combining the counts for these three compartments. Tumors with any number of CD8+ cells were considered as positive CD8+ T-cell infiltration.

RECQL deficient tumors with CD8+ T-cell infiltration within tumor cell nests (Table 1) or adjacent stroma (Supplementary Table 8) were highly significantly associated with larger tumors, high grade, de-differentiation, pleomorphism, higher mitotic index, high Ki67 expression, high risk Nottingham prognostic index (NPI), ER-, PR- and triple negative breast cancers (all *p* values ≤0.001) compared to RECQL proficient CD8- tumors. RECQL deficient tumors with CD8+ T-cell infiltration in distant stroma was significantly associated with pleomorphism and high-risk Nottingham prognostic index (NPI) (all *p* values ≤0.005) (Supplementary Table 9) compared to RECQL proficient CD8- tumors. When CD8+ T-cell infiltration was taken together (within tumor cell nest, adjacent and distant stroma), RECQL deficient tumors with total CD8+ T-cells were significantly associated with high grade, de-differentiation, pleomorphism, higher mitotic index, high Ki67 expression, high risk Nottingham prognostic index (NPI) and triple negative breast cancers (all *p* values ≤0.05) (Supplementary Table 9) compared to RECQL proficient CD8- tumors.

**Table 1:** Clinicopathological significance of RECQL and CD8 (within tumour cell nest) co-expression in breast cancers.

	RECQL-/CD8-	RECQL+/CD8+	RECQL+/CD8-	RECQL-/CD8+	X^2^
					*P*-value
**Tumour size**					
≤ 2cm	103 (54 %)	18 (49 %)	43 (56 %)	53 (42 %)	5.265 0.153
> 2cm	89 (46 %)	19 (51 %)	34 (44 %)	73 (58 %)	
**Tumour grade**					
1	33 (17 %)	5 (14 %)	18 (23 %)	9 (7 %)	35.766 <0.0001
2	70 (37 %)	9 (24 %)	38 (50 %)	32 (25 %)	
3	89 (46 %)	23 (62 %)	21 (27 %)	85 (68 %)	
**Tubule formation**					
1	11 (6 %)	2 (5 %)	5 (6 %)	4 (3 %)	19.923 0.003
2	70 (36 %)	7 (19 %)	36 (47 %)	29 (23 %)	
3	111 (58 %)	28 (76 %)	36 (47 %)	93 (74 %)	
**Pleomorphism**					
1	8 (4 %)	2 (5 %)	3 (4 %)	7 (6 %)	31.783 <0.0001
2	83 (43 %)	15 (41 %)	45 (58 %)	26 (20 %)	
3	101 (53 %)	20 (54 %)	29 (38 %)	93 (74 %)	
**Mitosis**					
1	59 (31 %)	11 (30 %)	38 (49 %)	25 (20 %)	36.8 <0.0001
2	47 (24 %)	2 (5 %)	17 (22 %)	19 (15 %)	
3	86 (45 %)	24 (65 %)	22 (29 %)	82 (65 %)	
**Histologic types**					
No special type (NST)	121 (63 %)	25 (68 %)	36 (47 %)	92 (73 %)	19.755 0.019
Lobular	15 (8 %)	4 (11 %)	11 (14 %)	13 (10 %)	
Other special types	6 (3 %)	2 (5 %)	2 (3 %)	2 (2 %)	
Mixed NST	50 (26 %)	6 (16 %)	28 (36 %)	19 (15 %)	
**Lymph node status**					
Absent	120 (63 %)	25 (68 %)	51 (66 %)	72 (57 %)	2.355 0.502
Present	72 (37 %)	12 (32 %)	26 (34 %)	54 (43 %)	
**Lymphovascular invasion**					
Absent	126 (66 %)	25 (68 %)	55 (71 %)	76 (60 %)	2.749 0.432
Present	66 (34 %)	12 (32 %)	22 (29 %)	50 (40 %)	
**Nottingham Prognostic index**					
Good prognostic group	58 (30 %)	11 (30 %)	33 (43 %)	20 (16 %)	29.345 <0.0001
Moderate prognostic group	110 (57 %)	17 (46 %)	42 (54 %)	79 (63 %)	
Poor prognostic group	24 (13 %)	9 (24 %)	2 (3 %)	27 (21 %)	
**ER status**					
Negative	49 (26 %)	14 (38 %)	9 (12 %)	52 (41 %)	22.959 <0.0001
Positive	142 (74 %)	23 (62 %)	68 (88 %)	74 (59 %)	
**PR status**					
Negative	80 (43 %)	17 (49 %)	18 (24 %)	76 (60 %)	26.444 <0.0001
Positive	107 (57 %)	18 (51 %)	58 (76 %)	50 (40 %)	
**Ki67 expression**					
Low≤14	68 (46 %)	6 (23 %)	44 (68 %)	27 (28 %)	29.128 <0.0001
High>14	79 (54 %)	20 (77 %)	21 (32 %)	68 (72 %)	
**Molecular subtypes by IHC**					
Luminal A	56 (38 %)	6 (22 %)	39 (59 %)	20 (18 %)	40.061 <0.0001
Luminal B	51 (34 %)	9 (32 %)	19 (29 %)	39 (35 %)	
Triple negative	32 (21 %)	9 (32 %)	6 (9 %)	37 (34 %)	
HER2 +	11 (7 %)	4 (14 %)	2 (3 %)	14 (13 %)	

Breast cancer specific survival (BCSS) was significantly shorter in RECQL deficient tumors with CD8+ T-cell infiltration within tumor cell nests (Fig. 2I, (*p* < 0.0001; HR = 0.87; 95 % CI =0.77-0.99), adjacent stroma (Supplementary Fig. 1A, *p =*0.038; HR = 0.87; 95 % CI =0.76-1.13), distant stroma (Supplementary Fig. 1B, p= 0.006; HR = 0.73; 95 % CI =0.59-0.90), and total CD8+ T-cell infiltration (Supplementary Fig. 1C, *p =* 0.04; HR = 0.86; 95 % CI =0.73-1.1) compared to RECQL proficient CD8- tumors. Taken together the data provides clinical evidence that in RECQL deficient tumors, CD8+ T-cell infiltration is associated with aggressive IBC.

**FOXP3+ T- cell infiltration in RECQL deficient BC:** T regulatory cells (Tregs) can inhibit antitumor responses and influence the activity of CD8+ TILs [11]. FOXP3, a member of the forkhead family of transcription factors, is restricted to specific population of Tregs [11]. We evaluated the associations between FOXP3+ T-cell infiltration and RECQL deficient IBC. RECQL deficient tumors with FOXP3+ T-cell infiltration within tumor cell nests (Table 2), within adjacent stroma (Supplementary Table 10), within distant stroma (Supplementary Table 10) and total FOXP3+ T-cells were all highly significantly associated with high grade, de-differentiation, pleomorphism, higher mitotic index, high Ki67 expression, high risk Nottingham prognostic index (NPI), ER-, PR- and triple negative breast cancers (all *p* values *p*≤0.0001).

**Table 2:** Clinicopathological significance of RECQL and FOXP3 (within tumour cell nest) co-expression in breast cancers.

	RECQL-/FOXP3-	RECQL+/FOXP3+	RECQL+/FOXP3-	RECQL-/FOXP3+	X^2^
					*P*-value
**Tumour size**					
≤ 2cm	227 (50 %)	9 (38 %)	81 (56 %)	41 (41 %)	7.208 0.066
> 2cm	230 (50 %)	15 (62 %)	63 (44 %)	60 (59 %)	
**Tumour grade**					
Grade 1	61 (13 %)	0 (0 %)	33 (23 %)	4 (4 %)	82.839 <0.0001
Grade2	171 (38 %)	3 (12 %)	66 (46 %)	12 (12 %)	
Grade 3	225 (49 %)	21 (88 %)	45 (31 %)	85 (84 %)	
**Tubule formation**					
1	19 (4 %)	0 (0 %)	11 (8 %)	2 (2 %)	40.438 <0.0001
2	164 (36 %)	2 (8 %)	60 (42 %)	14 (14 %)	
3	274 (60 %)	22 (92 %)	73 (50 %)	85 (84 %)	
**Pleomorphism**					
1	18 (4 %)	0 (0 %)	4 (3 %)	1 (1 %)	51.213 <0.0001
2	159 (35 %)	5 (21 %)	77 (53 %)	13 (13 %)	
3	280 (61 %)	19 (79 %)	63 (44 %)	87 (86 %)	
**Mitosis**					
1	155 (34 %)	3 (12 %)	74 (52 %)	9 (9 %)	93.34 <0.0001
2	107 (23 %)	0 (0 %)	28 (19 %)	10 (10 %)	
3	195 (43 %)	21 (88 %)	42 (29 %)	82 (81 %)	
**Histologic types**					
No special type (NST)	281 (62 %)	23 (96 %)	66 (46 %)	92 (91 %)	66.037 <0.0001
Lobular	51 (11 %)	0 (0 %)	23 (16 %)	0 (0 %)	
Other special types	16 (3 %)	0 (0 %)	6 (4 %)	0 (0 %)	
Mixed NST	109 (24 %)	1 (4 %)	49 (34 %)	9 (9 %)	
**Lymph node status**					
Absent	265 (58 %)	13 (54 %)	103 (72 %)	65 (64 %)	9.383 0.025
Present	192 (42 %)	11 (46 %)	41 (28 %)	36 (36 %)	
**Lymphovascular invasion**					
Absent	281 (62 %)	15 (63 %)	104 (72 %)	69 (68 %)	6.25 0.100
Present	176 (38 %)	9 (37 %)	40 (28 %)	32 (32 %)	
**Nottingham Prognostic index**					
Good prognostic group	120 (26 %)	3 (12 %)	65 (45 %)	16 (16 %)	48.613 <0.0001
Moderate prognostic group	265 (58 %)	11 (46 %)	70 (49 %)	60 (59 %)	
Poor prognostic group	72 (16 %)	10 (42 %)	9 (6 %)	25 (25 %)	
**ER status**					
Negative	106 (23 %)	14 (58 %)	22 (15 %)	61 (60 %)	79.867 <0.0001
Positive	350 (77 %)	10 (42 %)	121 (85 %)	40 (40 %)	
**PR status**					
Negative	181 (40 %)	12 (52 %)	41 (30 %)	70 (69 %)	41.115 <0.0001
Positive	269 (60 %)	11 (48 %)	98 (70 %)	31 (31 %)	
**Ki67 expression**					
Low≤14	154 (43 %)	1 (5 %)	69 (58 %)	15 (18 %)	43.358 <0.0001
High>14	206 (57 %)	18 (95 %)	49 (42 %)	68 (82 %)	
**Molecular subtypes by IHC**					
Luminal A	134 (35 %)	1 (5 %)	62 (52 %)	6 (7 %)	106.826 <0.0001
Luminal B	149 (39 %)	7 (35 %)	37 (31 %)	26 (28 %)	
Triple negative	59 (16 %)	10 (50 %)	15 (12 %)	50 (54 %)	
HER2 +	37 (10 %)	2 (10 %)	6 (5 %)	10 (11 %)	

BCSS was significantly shorter in RECQL deficient tumors with FOXP3+ T-cell infiltration within tumor cell nests (Fig. 2J, (*p =*0.002; HR = 0.87; 95 % CI =0.77-0.99) or adjacent stroma (Supplementary Fig. 2A, *p =*0.006; HR = 0.85; 95 % CI =0.74-0.97) or distant stroma (Supplementary Fig. 2B, p= 0.03; HR = 0.83; 95 % CI =0.71-0.98) or total FOXP3+ T-cell infiltration (Supplementary Fig. 1C, *p =* 0.006; HR = 0.8; 95 % CI =0.67-0.9) compared to RECQL proficient FOXP3- tumors.

**IL-17+ T- cell infiltration in RECQL deficient BC:** IL-17 is the signature cytokine of distinct CD4+ T helper 17 (Th17) cells [12]. The role of IL-17 in cancer is complex. During early carcinogenesis IL-17 can promote tumor formation, but in established tumors, IL-17 production by Th17 cells has been shown to promote antitumor immunity [12,13]. In breast cancer, IL-17 is associated with proliferation, invasion, metastasis and poor survival [14]. RECQL deficient tumors with IL-17+ T-cell infiltration within tumor nests alone (Supplementary Table 14), within adjacent stroma (Supplementary Table 15) and combined with adjacent stroma (Supplementary Table 16) were significantly associated with high grade, pleomorphism, higher mitotic index, high Ki67 expression high risk Nottingham prognostic index (NPI) (p≤0.001) and poor BCSS (Supplementary Fig. 3A, (*p =*0.007; HR = 0.83; 95 % CI =0.73-0.96), Supplementary Fig. 3B, (*p =*0.095; HR = 0.85; 95 % CI =0.71-1.0) and Supplementary Fig. 3C, (*p =*0.038; HR = 0.84; 95 % CI =0.72-0.98) respectively) compared to RECQL proficient IL-17- tumors.

**PDL1+ tumor cells or PDL1+ T cell infiltration in RECQL deficient IBC:** Programmed death ligand-1 (PDL1) and programmed death-1 (PD1) are key members of the PD pathway that is involved in immune regulation. The interaction of PDL1 with PD1 induces T cell suppression [15]. PDL1-PD1 targeting is an established immunotherapeutic approach in cancer. PDL1 is expressed in breast cancer cells [16] and in T-cells [16]. RECQL deficient tumors with tumor cell PDL1 expression (PDL1+) (Table 3) were significantly associated with high grade, pleomorphism, higher mitotic index, high Ki67 expression, lymph node positivity and high-risk Nottingham prognostic index (NPI) (all *p* values ≤0.001) compared to RECQL proficient PDL1- tumors. RECQL deficient tumors with PDL1 expression (PDL1+) TILs(Supplementary Table 17) were also significantly associated with high grade, de-differentiation, pleomorphism, higher mitotic index, high Ki67 expression, high-risk Nottingham prognostic index (NPI), ER-, PR-, triple negative tumors (all *p* values ≤0.0001) compared to RECQL proficient PDL1- tumors.

**Table 3:** Clinicopathological significance of RECQL and PDL1+ (tumours cells) co-expression in breast cancers.

	RECQL-/PDL1-	RECQL+/PDL1+	RECQL+/PDL1-	RECQL-/PDL1+	X^2^
					*P*-value
**Tumour size**					
≤ 2cm	43 (48 %)	61 (55 %)	4 (27 %)	197 (47 %)	5.050 0.168
> 2cm	47 (52 %)	50 (45 %)	11 (73 %)	222 (53 %)	
**Tumour grade**					
Grade 1	15 (17 %)	19 (17 %)	1 (7 %)	40 (9 %)	31.049 <0.0001
Grade2	39 (43 %)	47 (42 %)	11 (73 %)	129 (31 %)	
Grade 3	36 (40 %)	45 (41 %)	3 (20 %)	250 (60 %)	
**Tubule formation**					
1	4 (4 %)	8 (7 %)	0 (0 %)	18 (4 %)	5.303 0.506
2	27 (30 %)	41 (37 %)	6 (40 %)	127 (30 %)	
3	59 (66 %)	62 (56 %)	9 (60 %)	274 (66 %)	
**Pleomorphism**					
1	3 (3 %)	2 (2 %)	1 (6 %)	12 (3 %)	30.393 <0.0001
2	45 (50 %)	52 (47 %)	7 (47 %)	113 (27 %)	
3	42 (47 %)	57 (51 %)	7 (47 %)	294 (70 %)	
**Mitosis**					
1	33 (36 %)	47 (42 %)	9 (60 %)	105 (25 %)	26.682 <0.0001
2	25 (28 %)	23 (21 %)	2 (13 %)	89 (21 %)	
3	32 (36 %)	41 (37 %)	4 (27 %)	225 (54 %)	
**Histologic types**					
No special type (NST)	51 (57 %)	56 (51 %)	6 (40 %)	297 (71 %)	30.621 <0.0001
Lobular	11 (12 %)	14 (13 %)	5 (33 %)	31 (7 %)	
Other special types	3 (3 %)	5 (4 %)	1 (7 %)	12 (3 %)	
Mixed NST	25 (28 %)	36 (32 %)	3 (20 %)	79 (19 %)	
**Molecular subtypes by IHC**					
Luminal A	22 (33 %)	39 (42 %)	4 (33 %)	90 (25 %)	14.932 0.093
Luminal B	24 (37 %)	33 (36 %)	3 (25 %)	150 (42 %)	
Triple negative	14 (21 %)	17 (19 %)	4 (33 %)	84 (23 %)	
HER2 +	6 (9 %)	3 (3 %)	1 (9 %)	36 (10 %)	
**Lymph node status**					
Absent	66 (73 %)	77 (69 %)	9 (60 %)	225 (54 %)	17.464 0.001
Present	24 (27 %)	34 (31 %)	6 (40 %)	194 (46 %)	
**Lymphovascular invasion**					
Absent	64 (71 %)	76 (69 %)	8 (53 %)	250 (60 %)	6.512 0.089
Present	26 (29 %)	35 (31 %)	7 (47 %)	169 (40 %)	
**Nottingham Prognostic index**					
Good prognostic group	33 (37 %)	49 (44 %)	5 (33 %)	81 (19 %)	40.855 <0.0001
Moderate prognostic group	52 (58 %)	47 (42 %)	8 (54 %)	252 (60 %)	
Poor prognostic group	5 (5 %)	15 (14 %)	2 (13 %)	86 (21 %)	
**ER status**					
Negative	22 (25 %)	22 (20 %)	5 (33 %)	131 (31 %)	6.484 0.090
Positive	67 (75 %)	89 (80 %)	10 (67 %)	288 (69 %)	
**PR status**					
Negative	39 (45 %)	29 (27 %)	10 (67 %)	191 (46 %)	16.215 0.001
Positive	48 (55 %)	78 (73 %)	5 (33 %)	223 (54 %)	
**Ki67 expression**					
Low≤14	27 (46 %)	44 (49 %)	6 (50 %)	113 (33 %)	11.074 0.011
High>14	32 (54 %)	45 (51 %)	6 (50 %)	231 (67 %)	

BCSS was significantly shorter in RECQL deficient tumors with tumor cell PDL1+ (Fig. 2K, (*p =*0.0003; HR = 0.73; 95 % CI =0.57-0.94) or PDL1+ TILs (Supplementary Fig. 3A, *p* <0.0001; HR = 0.26; 95 % CI =0.10-0.65) compared to RECQL proficient PDL1- tumors.

**PD1+ T cell infiltration in RECQL deficient IBC:** PD1 is an inhibitory receptor expressed by all T cells (including Tregs) and regulates T cell effector functions in tumor microenvironment. PD1 can limit the activation and function of CD8^+^ T cells in cancers [15]. RECQL deficient tumors with PD1+ T-cell infiltration (Table 4) were significantly associated with high grade, de-differentiation, pleomorphism, higher mitotic index, high Ki67 expression, high risk Nottingham prognostic index (NPI), ER-, PR- and triple negative breast cancers (all *p* values ≤0.0001) compared to RECQL proficient FOXP3- tumors. BCSS was not significant (Supplementary Fig. 4B, *p =* 0.07; HR = 0.88; 95 % CI =0.74–1.0) in RECQL deficient tumors with PD1+ T-cell infiltration compared to RECQL proficient CD8- tumors.

**Table 4:** Clinicopathological significance of RECQL and PD1+ (TILs) co-expression in breast cancers.

	RECQL-/PD1-	RECQL+/PD1+	RECQL+/PD1-	RECQL-/PDL1+	X^2^
					*P*-value
**Tumour size**					
≤ 2cm	104 (50 %)	31 (46 %)	44 (63 %)	159 (46 %)	7.22 0.065
> 2cm	103 (50 %)	37 (54 %)	26 (37 %)	188 (54 %)	
**Tumour grade**					
Grade 1	41 (20 %)	5 (7 %)	16 (23 %)	26 (7 %)	59.089 <0.0001
Grade2	85 (41 %)	26 (38 %)	34 (49 %)	96 (28 %)	
Grade 3	81 (39 %)	37 (55 %)	20 (28 %)	225 (65 %)	
**Tubule formation**					
1	19 (9 %)	2 (3 %)	7 (10 %)	5 (1 %)	34.291 <0.0001
2	79 (38 %)	24 (35 %)	26 (37 %)	96 (28 %)	
3	109 (53 %)	42 (62 %)	37 (53 %)	246 (71 %)	
**Pleomorphism**					
1	10 (5 %)	2 (3 %)	1 (1 %)	6 (2 %)	89.639 <0.0001
2	97 (47 %)	16 (23 %)	49 (70 %)	76 (22 %)	
3	100 (48 %)	50 (74 %)	20 (29 %)	265 (76 %)	
**Mitosis**					
1	82 (40 %)	23 (34 %)	35 (50 %)	80 (23 %)	48.374 <0.0001
2	53 (25 %)	10 (15 %)	17 (24 %)	63 (18 %)	
3	72 (35 %)	35 (51 %)	18 (26 %)	204 (59 %)	
**Histologic types**					
No special type (NST)	110 (53 %)	44 (65 %)	30 (43 %)	270 (78 %)	60.986 <0.0001
Lobular	24 (12 %)	8 (12 %)	10 (14 %)	21 (6 %)	
Other special types	11 (5 %)	2 (3 %)	7 (10 %)	3 (1 %)	
Mixed NST	62 (30 %)	14 (20 %)	23 (33 %)	53 (15 %)	
**Lymph node status**					
Absent	124 (60 %)	42 (62 %)	54 (77 %)	198 (57 %)	9.897 0.019
Present	83 (40 %)	26 (38 %)	16 (23 %)	149 (43 %)	
**Lymphovascular invasion**					
Absent	138 (67 %)	41 (60 %)	55 (79 %)	210 (60 %)	9.331 0.025
Present	69 (33 %)	27 (40 %)	15 (21 %)	137 (40 %)	
**Nottingham Prognostic index**					
Good prognostic group	65 (31 %)	18 (26 %)	35 (50 %)	66 (19 %)	38.292 <0.0001
Moderate prognostic group	117 (57 %)	40 (59 %)	31 (44 %)	208 (60 %)	
Poor prognostic group	25 (12 %)	10 (15 %)	4 (6 %)	73 (21 %)	
**ER status**					
Negative	38 (18 %)	24 (36 %)	6 (9 %)	135 (39 %)	43.105 <0.0001
Positive	169 (82 %)	43 (64 %)	63 (91 %)	211 (61 %)	
**PR status**					
Negative	78 (38 %)	26 (40 %)	20 (29 %)	188 (55 %)	25.655 <0.0001
Positive	127 (62 %)	39 (60 %)	49 (71 %)	153 (45 %)	
**Ki67 expression**					
Low≤14	80 (47 %)	21 (37 %)	33 (61 %)	81 (30 %)	25.596 <0.0001
High>14	90 (53 %)	36 (63 %)	21 (39 %)	191 (70 %)	
**Molecular subtypes by IHC**					
Luminal A	73 (41 %)	18 (31 %)	29 (52 %)	60 (21 %)	53.87 <0.0001
Luminal B	70 (39 %)	17 (29 %)	21 (37 %)	108 (37 %)	
Triple negative	25 (14 %)	19 (33 %)	5 (9 %)	89 (30 %)	
HER2 +	11 (6 %)	4 (7 %)	1 (2 %)	36 (12 %)	

We also correlated expression of RECQL with immune cell infiltration. As shown in Supplementary Table 18, there was a significant inverse correlation between RECQL expression and PD1+ (*p*=0.004) or IL-17+ immune cell infiltration (p=0.01).

**Multivariate analysis for survival:** In multivariate analysis, we observed that RECQL, CD8 and FOXP3 were independently associated with BCSS (Supplementary Table 19). Larger tumor size and positive lymph node status were other parameters independently associated with survival in this analysis (Supplementary Table 19).

**Fig. 3:**
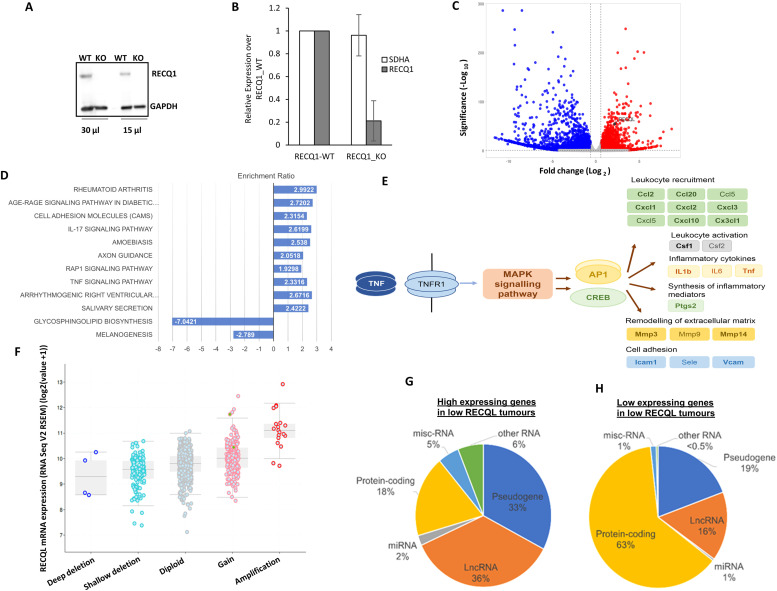
RECQL depletion and gene expression analysis in MDA-MB-231 cells A. Isogenic MDA-MB-231 RECQL knockout (RECQL-KO) and its wild-type control (RECQL-WT) cells were generated using the CRISPR/Cas9 system and validated by immunoblot detection of RECQL in total cell lysates. RECQL protein levels were measured in two sample volumes. GAPDH is used as a loading control. B. Measuring the RECQL mRNA levels by RT-qPCR normalized to GAPDH. SDHA served as a second housekeeping gene. Relative expressions of RECQL and SDHA is shown here. C. Volcano plot obtained from differentially gene expression (DGE) analysis (fold change (≥±1) combined with adjusted *p*-value (<0.05) comparing genes associated with low versus high *RECQL* mRNA expression. D. Over representation KEGG pathway analysis for genes higher or lower in RECQL KO cells compared to WT cells. Pathways shown with positive enrichment ratio are expressed higher in RECQL KO and pathways with negative enrichment ratio are expressed lower in RECQL KO. All pathways are FDR corrected p-value <0.05, from genes that were significantly differentially expressed log2FC ≥1 and FDR p value <0.05. E. Representation of the genes that were enriched in the TNF (hsa04668) and IL17 (hsa04657) pathways highlighting the higher levels of TNFα and transcription factors AP1 and CREB, leading to increase in cytokine and chemokine signalling in RECQL KO cells. Genes shown in bold were expressed significantly higher level in RECQL KO cells compared to WT (log2FC ≥1 and FDR p value <0.05). F. Comparison of RECQL gene expression to copy number variation in TCGA-BRCA (*n =* 960). GISTIC analysis shows changes in *RECQL* mRNA levels in tumors with copy number variations. The expression data was from normalized illumina HiSeq RNA-Seq data. The copy number variations are deep deletions (>2 copies deleted), shallow deletion (few copies altered), diploid, gains (few copies gained), amplification (>2 copies gained). Pearson correlation R = 0.49, p < 0.001. RNA gene types (Ensembl MART) are shown for non-coding RNAs (lncRNA, all pseudogenes, miRNAs and other RNA which include snoRNA, tRNA and MT-RNA) plus protein-coding genes, as percentages in the pie charts for G) RNAs expressed higher in low *RECQL* tumors (*n =* 9277 confirmed gene types) and H) RNAs expressed lower in low *RECQL* tumors (*n =* 451 confirmed gene types).

**Gene expression profiling in RECQL knock-out (KO) MDA-MB-231 cells:** Immunohistochemical analysis presented above shows that RECQL low tumors with T-cells infiltration (CD8+, FOXP3+, IL17, PDL1+, or PD1) are associated with triple negative breast cancers. Pre-clinically, in a triple negative breast cancer cell line (MDA-MB-231), we generated isogenic RECQL- wildtype (WT) and RECQL-knock-out (KO) clones using a CRISPRS/Cas-9 system (Fig. 3A and B). Total RNA was extracted from MDA-MB-231 RECQL-WT and RECQL-KO clones and subjected to RNA seq analysis. Over representation KEGG pathway analysis for genes higher or lower in RECQL-KO cells compared to WT cells is shown in Fig. 3C. Interestingly, we observed enrichment of TNFα (hsa04668) and IL17 (hsa04657) pathway genes (Fig. 3D and E). The data suggests that higher levels of TNFα and transcription factors AP1 and CREB may lead to an increase in cytokine and chemokine signaling in RECQL-KO MDA-MB-231cells.

Taken together, the clinical and pre-clinical data provides strong evidence that RECQL deficient tumors have frequent T-cell infiltration which is associated with aggressive pathology and poor survival.

### Genomic and transcriptomic analysis of RECQL in BC-TCGA

Pre-clinically, utilizing an unbiased integrative genomics approach, we have observed that expression of *ESR1*, the gene encoding ERα, is directly activated by RECQL. More than 35 % of RECQL binding sites were co-bound by ERα genome-wide [8]. Mechanistically, RECQL cooperates with FOXA1, the pioneer transcription factor for ERα, to enhance chromatin accessibility at the *ESR1* regulatory regions in a helicase activity-dependent manner [8]. Given the potential role for RECQL in transcriptional regulation, we speculated that RECQL deficiency in breast cancer will not only promote genomic instability but will also lead to global transcriptomic alterations that could adversely influence its pathology. To address this possibility, we conducted genomic and transcriptomic analysis of tumor samples from patients with invasive breast cancers (IBC) in the TCGA (the cancer genome atlas) cohort [17].

First, we utilized cBioportal to examine mutations and copy number variations of the *RECQL* gene in the BC-TCGA firehose legacy cohort (1101 patients). Interestingly, only 25/963 patients (2.5 %) showed alterations where the majority were *RECQL* amplification. Only 2 missense mutations were identified (L286Q and P535S). Correlation between copy number variation and gene expression showed significant positive correlation (Fig. 3F; n=960 Pearson 0.49, *p* < 0.001). We evaluated DNA methylation status within the transcription start site (TSS) CpG island using SMART app, which utilizes UCSC Xena datasets correlating beta values (Illumina Infinium 450K methylation chip) and gene expression values (RNA-Seq dataset). The majority of the CpG island probes within the TSS were unmethylated and showed significant weak negative correlation with gene expression. The most internal CpG site (cg5389560) varied greatly in methylation status (beta value 0-0.8) but still only showed weak positive correlation with gene expression (R = 0.16, *p* <0.01; Supplementary Fig. 5A and B). Therefore, DNA methylation is not linked with low expression of RECQL in IBC.

Next, we investigated if RECQL levels can influence global gene expression. Differential gene expression between low *RECQL* expressing tumors and high *RECQL* tumors was compared in the BC-TCGA RNA-Seq dataset (Supplementary Table 20). High expression of 10061 genes and low expression of 477 genes was observed in low RECQL tumors (Supplementary Table 20). Interestingly, among the high expressing genes in low RECQL tumors, only 18 % are protein-coding genes (Fig. 3G). In contrast to the genes expressed lower in RECQL low tumors, whichwas 63 % (Fig. 3H). This suggests that RECQL low tumors were associated with a higher expression of lncRNAs and pseudogenes, which is feature of increased genomic instability [18,19]. Gene enrichment analysis identified significant KEGG pathways (FDR p-value <0.05; top five pathways shown Supplementary Fig. 5C). Interestingly, lower expression of integrins (log2 fold changes - *ITGA4* -1.273, *ITGB3* -1.244, *ITGB1* -1.229) were the main genes within the pathways. PI3K-Akt (PKB) signaling pathway highlighted lower expression of PI3K subunits and higher expression of HRas (log2 fold change1.2713).

**Tumor mutation burden (TMB) and homologous recombination deficiency (HRD) in breast cancers with RECQL germ-line mutations:** The bioinformatic data in RECQL low sporadic breast cancers suggest a genomic instability phenotype. To validate these observations further, we conducted genomic analysis in five breast cancer patients with RECQL germ-line deficiency. In the five carriers of RECQL germ-line mutations predicted to abolish its helicase activity [5], we performed whole genome sequencing (WGS) on matched tumor and germline DNA. Whole exome sequencing (WES) was completed in tumor samples alone. We used matched tumor normal WGS and WES data for assessing microsatellite instability (MSI) and tumor mutational burden (TMB). Tumor only WGS data was utilized for homologous recombination deficiency (HRD) analysis. The data is summarized in Supplementary Table 21. All WGS data were suitable for MSI and HRD analysis. Only one tumor sample (patient 3) had a high HRD score of 59, likely due to an oncogenic CDK12 mutation (NP_057591.2:p.Arg1067Ter) found in this tumor which is associated with HRD [20]. All tumor samples had a low microsatellite (MS) instability (MSI-L) with unstable MS proportion of around or over 20 %. One tumor sample (patient 5) had a high TMB (>15) and three had an intermediate TMB score of around 10. The tumour (patient 3) with high HRD score had a low TMB (5.4). Taken together, the data provides evidence that germline *RECQL* deficiency may contribute to genomic instability with an increased TMB phenotype.

## Discussion

RECQL helicase is essential for the maintenance of replication fork progression, recombination, and DNA repair [2]. RECQL loss is therefore expected to increase genomic instability and promote a mutator phenotype [21] leading to increased risk of cancer. In the current study we not only provide the first clinical evidence that RECQL loss may be an early event during breast cancer pathogenesis, but also show that in established invasive breast cancers, RECQL deficiency is associated with immune cell infiltration, aggressive pathology, and poor prognosis.

The incidence of pre-invasive breast DCIS continues to increase [22]. Although surgery (mastectomy or wide local excision), with or without adjuvant radiotherapy are the main treatment modalities, personalization of DCIS therapy is an area of unmet need. Whilst a subset of low-grade DCIS may never progress to invasive cancer, a proportion of high-grade DCIS, despite surgery and adjuvant radiotherapy, may still recur [22]. Therefore, development of biomarkers of aggressive phenotype is highly desirable. Emerging data suggest that aggressive DCIS may result from the accumulation of somatic mutations [23]. We speculated that RECQL loss may influence the development of high-grade DCIS. In the current study, we provide the first clinical evidence that RECQL loss is a feature of some DCIS which is associated with aggressive phenotype and adverse survival outcomes. We have previously shown that loss of key base excision repair (BER) repair proteins such as XRCC1 [24] or polymerase β [25] in DCIS are also linked with aggressive clinicopathological features and survival.

In established IBC, the complex tumor microenvironment may include infiltrating immune cells. We and others have shown that CD8^+^ T lymphocytic infiltration are associated with high tumor grade, hormone receptor negative, and basal-like phenotype tumors [26,27]. Moreover, high total CD8^+^ counts promote better survival outcomes [26,27]. Although mechanisms of immune cell infiltration are multifactorial, impaired tumor cell DNA repair with associated genomic instability will increase mutagenicity, increase neoantigen load on tumor cell surface and enhance immunogenicity. Previously, we have shown that low RECQL breast tumors were significantly associated with low PARP1, BRCA1 negative, low RAD51, low ATM, low nuclear pChk1, low nuclear Chk2, low XRCC1, low FEN1, low SMUG1, and low DNA-PKcs expression [6] . Moreover, low RECQL tumors were also significantly associated with low levels of other RecQ helicases, including RECQL4, BLM, and WRN [6]. Together, the data supports the view that low RECQL tumors have features of genomic instability associated with low expression of multiple DNA repair proteins [6]. In the current study, we have shown for the first time that RECQL low tumors with increased CD8+, FOXP3+, PDL1+ or PD1+ TILs are not only associated with aggressive phenotype but also with adverse survival outcomes. In a recent study, we have also shown that breast tumors that expressed low XRCC1 are also associated with high CD8^+^ TILs counts, aggressive phenotype and reduced poor survival. Importantly, PD1^+^ or PDL1^+^ breast cancers with low XRCC1 were linked to aggressive cancers and reduced survival including in ER^–^ breast cancers in that study [10]. BRCA1 and BRCA2 proteins perform critical functions during homologous recombination. Mutations within the BRCA genes lead to impaired DNA repair and an increased risk of early-onset breast cancer. BRCA-mutated breast tumors are also characterized by the presence of TILs such as CD4+, CD8+, and FOXP3+ T lymphocytes [28]. In contrast, BRCA-mutated breast cancers with increased TILs are associated with better survival outcomes [28]. Similarly, loss of MMR genes such as MLH-1, PMS-2, MSH-2 and MSH-6 leads to MSI and increases the risk of colorectal cancers (CRC). CRCs with MSI have increased TMB, increased TILs and better survival outcomes [29]. In contrast to BRCA and MMR studies, our data shows that RECQL low tumors with immune cell infiltration have poor survival outcomes. Although the reason for this observation is unknown, we speculate that different DNA repair deficient backgrounds could influence different subsets of T -cell infiltration or tumor cell biology in different DNA repair deficiency states itself could influence outcomes. An intriguing pre-clinical observation in the current study was that RNA seq analysis in RECQL _KO_ MDA-MB-231 cells revealed enrichment of chemokine and cytokine gene expression. Whilst altered tumor cytokine/chemokine profile could influence the type of TIL subsets, detailed mechanistic investigations will be required to address this possibility.

In the IBC-TCGA cohort, low *RECQL* tumors had a higher expression of lncRNAs and pseudogenes, which is feature of increased genomic instability [18,19]. For additional validation, we exome and genome sequenced breast cancer from five patients who were RECQL germ-line mutation carriers. We observed intermediate to high TMB in 4/5 tumors and HRD phenotype in 1/5 tumors. High TMB was also reported in a colon tumor sample from a patient with a germline pathogenic mutation [30]. Taken together, the data supports the view that RECQL deficiency in breast cancer leads to genomic instability and immune infiltration. We conclude that RECQL based stratification in breast cancer is feasible for immune-oncology approaches.

## Patients and methods

Two cohorts of invasive breast cancer (*n*=1600) and DCIS (*n*=776) diagnosed and treated at City Hospital, Nottingham, United Kingdom from 1987 to 2013 were used in this retrospective study. All the samples were arranged in tissue microarray (TMA) as previously described.

For the DCIS cohort, demographic data and histopathological parameters were recorded, including age at diagnosis, tumor size, grade, diagnostic method (screening or symptomatic), presence of comedo necrosis, adjuvant radiotherapy (RT) and local recurrence-free survival (LRFS) based on the time (months). A positive tumor margin was subjected to re-excision after the breast conserving surgery (BCS), on the assumption that the first six months after BCS was not considered as a recurrence. Tumors that develop a contralateral side were not considered as recurrence. Molecular classification based on hormonal receptor expression (Estrogen Receptor (ER) and Progesterone Receptor (PR)), HER2 status, and Ki-67 proliferation index was available. A summary of demographic data is summarized in (Supplementary Table 1). In terms of the invasive BC cohort, clinical and tumor characteristics including patient's age at diagnosis, histologic tumor type, grade, tumor size, lymph node status, Nottingham Prognostic Index (NPI), and lymphovascular invasion (LVI) were available (Supplementary Table 5). All samples in the study series were constructed in TMAs using a 3D Histech® Grand Master® machine and 1mm cores.

**RECQL protein expression:** The assessment of the expression of RECQL protein in invasive BCSS and DCIS by immunohistochemistry (IHC) was conducted using the Novocastra Novolink™ Polymer Detection Systems kit (Code: RE7280-K, Leica, Biosystems, Newcastle, UK). 4 μm thick TMA sections were dewaxed and endogenous peroxidase activity was blocked with 0.3 % hydrogen peroxide in methanol for 10 min. Antigen retrieval was performed in citrate buffer pH 6.0 using a microwave (Whirlpool JT359 Jet Chef 1000 W) for 20 min. RECQL antibody (Bethyl Laboratories, catalog no. A300-450A) was used at a dilution of 1:1,000 for 60 minutes. The sections were counterstained with hematoxylin. For each run, negative and positive (by omission of the primary antibody and IgG-matched serum) controls were included in each run. The negative control ensured that all the staining was produced from the specific interaction between antibody and antigen.

RECQL expression was assessed using the percentage and intensity of the expression and H-score (semi-quantitative histochemical scoring) was calculated as previously described [6]

### CD8, FOXP3, IL17, PDL1 and PD1 immunohistochemistry (IHC) in IBC

TMAs were immunohistochemically profiled for CD8, FOXP3, PDL1, PD1 and other biological antibodies [26]. Supplementary Table 6 summarizes protocols, antibody dilution, scoring methodology. as previously described [6,10,24]. Immunohistochemical staining was performed using the Thermo Scientific Shandon Sequenza chamber system (REF: 72110017), in combination with the Novolink Max Polymer Detection System (RE7280-K: 1250 tests) as described in previous publications [26,31], and the Leica Bond Primary Antibody Diluent (AR9352), each used according to the manufacturer's instructions (Leica Microsystems). The number of CD8+ T lymphocytes was counted in three locations in each tumor: intertumoral compartment (within the tumor cell nests), within the distant stroma (defined as > one tumor cell diameter away from the tumor), and within the adjacent stroma (defined as CD8+ cells within one tumor cell diameter of the tumor). The total number of CD8+ T cells was determined by combining the counts for these three compartments. FOXP3, IL17, PD1 and PDL1 positive T lymphocytes were similarly assessed. Not all cores within the TMA were suitable for IHC assessments as some cores were missing or contained inadequate invasive cancer (<15 % tumor).

**Statistical analysis:** All statistical analysis was conducted with IBM SPSS software v26 (Chicago, IL, USA). A two-sided *p. value* <0.05 was considered statistically significant. To correlate RECQL protein level and clinicopathological factors in the invasive BC and primary DCIS series, Crosstabs chi-square test was used after dichotomizing RECQL protein level into high and low based on the X-tile value (X-tile software version 3.6.1, copyright Yale University 2003–05). An H score of ≥215 was taken as the cut-off for high RECQL level. Continuous data analysis was carried out using the Mann-Whitney U test and Kruskal-Walli's test. RECQL1 was combined with CD8, FOXP3, IL17, PDL1 and PD1 to assess the impact of their co-expression on the clinicopathological parameters of breast cancer. Univariate and multivariate statistical analysis and Kaplan-Meier curves with patients’ outcomes based on LRFI were performed on the pure DCIS series and based on BCSS in the invasive BC cohort.

### Bioinformatics analysis

CBioportal was performed on the Breast invasive carcinoma TCGA firehose legacy cohort (1101 patients) to identify mutations and copy number variations for the *RECQL* gene [32]. The BRCA (TCGA breast cancer) cohort RNA expression data was analyzed. The data was obtained from GDC (https://portal.gdc.cancer.gov/). The RNA-seq data (specimens n=1090) were firstly ranked (lowest to highest expression) for *RECQL*, then data split into quartiles. Differentially expressed genes between Q1 and Q4 were identified using DESeq2 [33]. Differential genes obtained significant change of log2 fold of 1 and above, FDR-*p* value <0.05. Gene set enrichment analysis was performed using WebGestalt with significant KEGG pathways shown (FDR-p value <0.05) [34]. Using the SMART app, we correlated DNA methylation beta values (from the Infinium 450methylation array) with RNA expression data (RNA-seq) [35].

### RNA isolation and RT-qPCR

Isogenic MDA-MB-231 RECQL-knock-out (KO) and its wild-type control (RECQL-WT) cells were generated using the CRISPR/Cas9 system [36]). Total RNA was extracted using the TRIzol reagent (Invitrogen) according to the manufacturer's instructions. A total of 0.5 μg of RNA was used for reverse transcription using the iScript Reverse Transcription Supermix kit (Bio-Rad) according to the manufacturer's instructions. The cDNA was subjected to real-time quantitative PCR using iTaq Universal SYBR Green Supermix (Bio-Rad) in triplicate. Reactions were cycled at 95 °C for 30 s; followed by 40 cycles of 94 °C for 10 s and 60 °C for 15s with fluorescence data collection during the anneal/extension step on the CFX96 Real-Time PCR System (Bio-Rad). The relative transcript levels were normalized to the housekeeping gene *GAPDH* and differential expression was measured using the 2-ΔΔCT method. The housekeeping gene *SDHA* served as a negative control in RT-qPCR experiments. The RECQL primers are Forward 5′-CAATGGCTGGAAAGGAGGTA-3′; Reverse 5′-CAGAGTTAAAAGCAGCCCTGGT-3′.

### RNA-seq analysis

Total RNA from MDA-MB-231 RECQL-WT and RECQL-KO clones was extracted using the RNeasy plus micro kit (Qiagen). RNA integrity was checked with a Bioanalyzer (Agilent), and only samples with an RNA integrity number (RIN) of >9.5 were subsequently subjected to mRNA-seq. The mRNA-seq samples were pooled and sequenced on HiSeq using Illumina TruSeq mRNA Prep Kit RS-122-2101 and paired-end sequencing. The samples had ∼79–101 million pass filter reads with a base call quality of above ∼90 % of bases with Q30 and above. Reads of the samples were trimmed for adapters and low-quality bases using Trimmomatic software before alignment with the reference genome (Human - hg19) and the annotated transcripts using STAR. The average mapping rate of all samples was ∼95 %. Unique alignment is above 89 %. The mapping statistics are calculated using Picard software. The samples had ∼0.88 % ribosomal reads. Percent coding bases were between 64-66 %. Percent UTR bases are 29–31 %, and mRNA bases were between 93-94 % for all the samples. Library complexity was measured in terms of unique fragments in the mapped reads using Picard's MarkDuplicate utility. The samples have 64–70 % non-duplicate reads.

Read count per gene was calculated by HTSeq under the annotation of Gencode and normalized by size factor implemented in the *DESeq2* package. Regularized logarithm transformation (rlog) values of gene expression were used to perform hierarchical clustering and principal component analysis. To assess differential gene expression between different conditions (e.g., constructs vs. mocks), we used a generalized linear model within *DESeq2* that incorporates information from counts and uses negative binomial distribution with fitted mean and a gene-specific dispersion parameter. *DESeq2* used Wald statistics for significance testing and the Benjamini-Hochberg adjustment for multiple corrections.

### Western blotting

Cells were harvested after washing with phosphate-buffered saline (PBS) and whole cell lysates were prepared using radioimmunoprecipitation assay (RIPA) buffer containing protease inhibitor cocktail (Roche) and subjected to immunoblot detection of RECQL using anti-RECQL (Bethyl lab) antibody.

### Tumour and germline DNA analysis

Whole genome of matched germline and tumour DNA from five patients with the French-Canadian founder RECQL mutation (c.634C>T, p.Arg215*) were sequenced 20x mean depth of coverage). IDT library kit was used for the library preparation and NoVaseq 6000 was used for sequencing. Whole exome sequencing was done for the tumour DNA with a higher depth of coverage (200x) to have a better estimation of TMB. Dragen Somatic software version 4.0.3 from Illumina Inc. was used for analyzing the matched sequence data for determining TMB, MSI and HRD scores.

## Data availability statement

Data supporting the study can be found in the supplementary information file, and the corresponding author can make any materials available upon request. Aggregate data from the referenced datasets are available from the corresponding author on reasonable request. Primary datasets generated during the study are available in supplementary Table 19.

## CRediT authorship contribution statement

**Ayat Lashen:** Data curation, Formal analysis, Investigation, Methodology, Writing – original draft, Writing – review & editing. **Abdulbaqi Al-Kawaz:** Data curation, Formal analysis, Investigation, Methodology, Writing – original draft, Writing – review & editing. **Jennie N Jeyapalan:** Conceptualization, Data curation, Formal analysis, Software, Visualization, Writing – original draft, Writing – review & editing. **Shatha Alqahtani:** Data curation, Formal analysis, Investigation, Methodology, Writing – original draft, Writing – review & editing. **Ahmed Shoqafi:** Data curation, Formal analysis, Investigation, Methodology, Writing – original draft, Writing – review & editing. **Mashael Algethami:** Data curation, Formal analysis, Investigation, Methodology, Writing – original draft, Writing – review & editing. **Michael Toss:** Data curation, Formal analysis, Investigation, Methodology, Writing – original draft, Writing – review & editing. **Andrew R Green:** Data curation, Formal analysis, Investigation, Methodology, Writing – original draft, Writing – review & editing. **Nigel P Mongan:** Data curation, Formal analysis, Investigation, Methodology, Writing – original draft, Writing – review & editing. **Sudha Sharma:** Data curation, Formal analysis, Investigation, Methodology, Writing – original draft, Writing – review & editing. **Mohammad R Akbari:** Data curation, Formal analysis, Investigation, Methodology, Writing – original draft, Writing – review & editing. **Emad A Rakha:** Data curation, Formal analysis, Investigation, Methodology, Writing – original draft, Writing – review & editing. **Srinivasan Madhusudan:** Conceptualization, Data curation, Formal analysis, Funding acquisition, Investigation, Methodology, Project administration, Resources, Software, Supervision, Validation, Visualization, Writing – original draft, Writing – review & editing.

## Declaration of Competing Interest

The authors declare that they have no known competing financial interests or personal relationships that could have appeared to influence the work reported in this paper.

## Appendix Supplementary materials

Image, application 1

Image, application 2

Image, application 3
