# Supplementary material for: Immune infiltration, aggressive pathology, and poor survival outcomes in RECQL helicase deficient breast cancers
Source: Neoplasia. 2023 Dec 21;47:100957. doi: 10.1016/j.neo.2023.100957 (PMC10777014; doi:10.1016/j.neo.2023.100957)
Supplement: Supplementary file 1 [file mmc1.docx]

**Supplementary Figures**


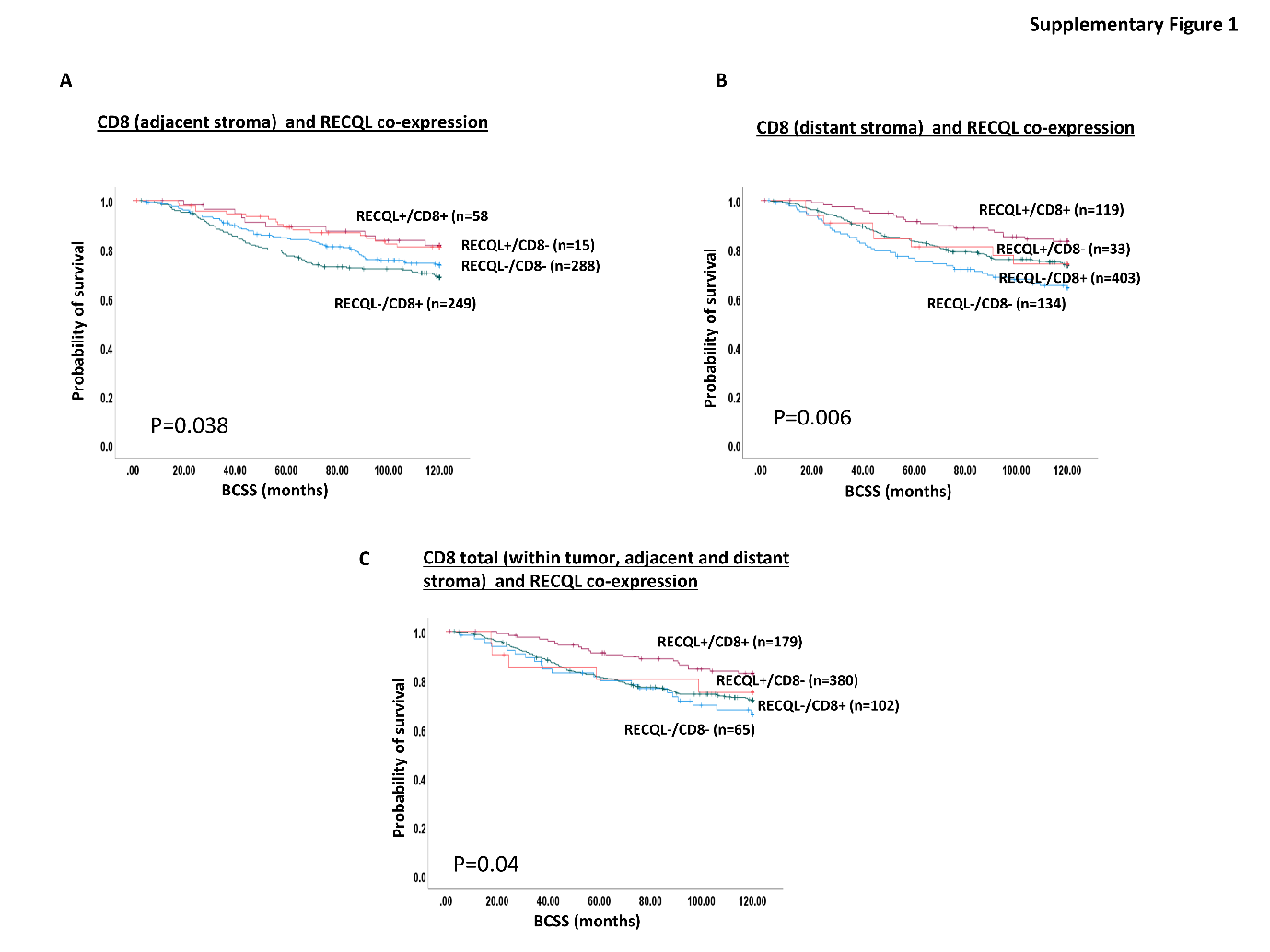


**Supplementary Figure 1: A.** Kaplan Meir curve shows the association between CD8 (adjacent stroma) and RECQL co-expression with breast cancer specific survival (BCSS) **B.** Kaplan Meir curve shows the association between CD8 (distant stroma) and RECQL co-expression with BCSS. **C.** Kaplan Meir curve shows the association between CD8 total (within tumour, adjacent and distant stroma) and RECQL co-expression with BCSS.


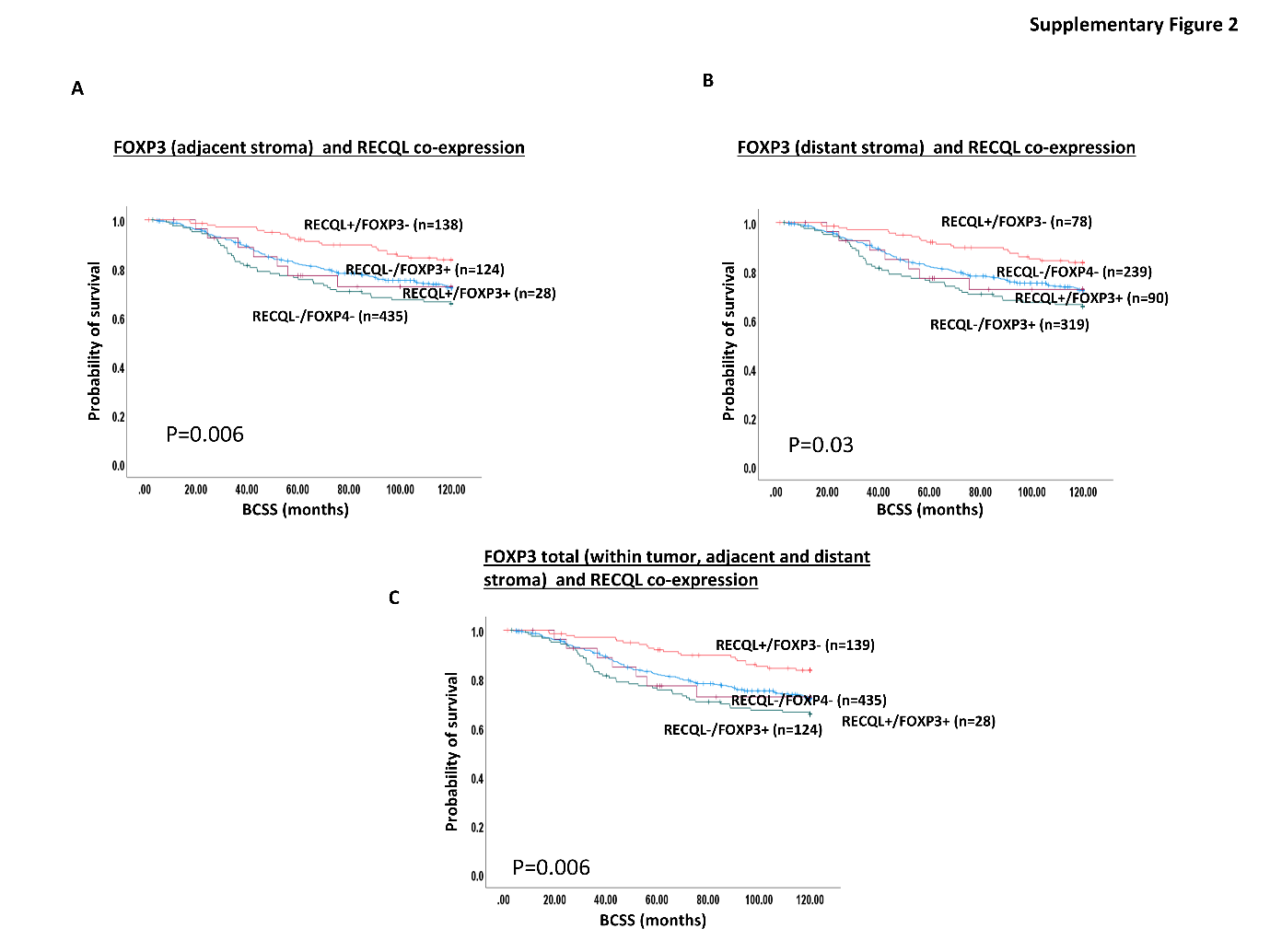


**Supplementary Figure 2.** A: Kaplan Meir curve shows the association between FOXP3 (adjacent stroma) and RECQL co-expression with BCSS. **B.** Kaplan Meir curve shows the association between FOXP3 (distant stroma) and RECQL co-expression with BCSS. **C.** Kaplan Meir curve shows the association between FOXP3 total (within tumour, adjacent and distant stroma) and RECQL co-expression with BCSS.


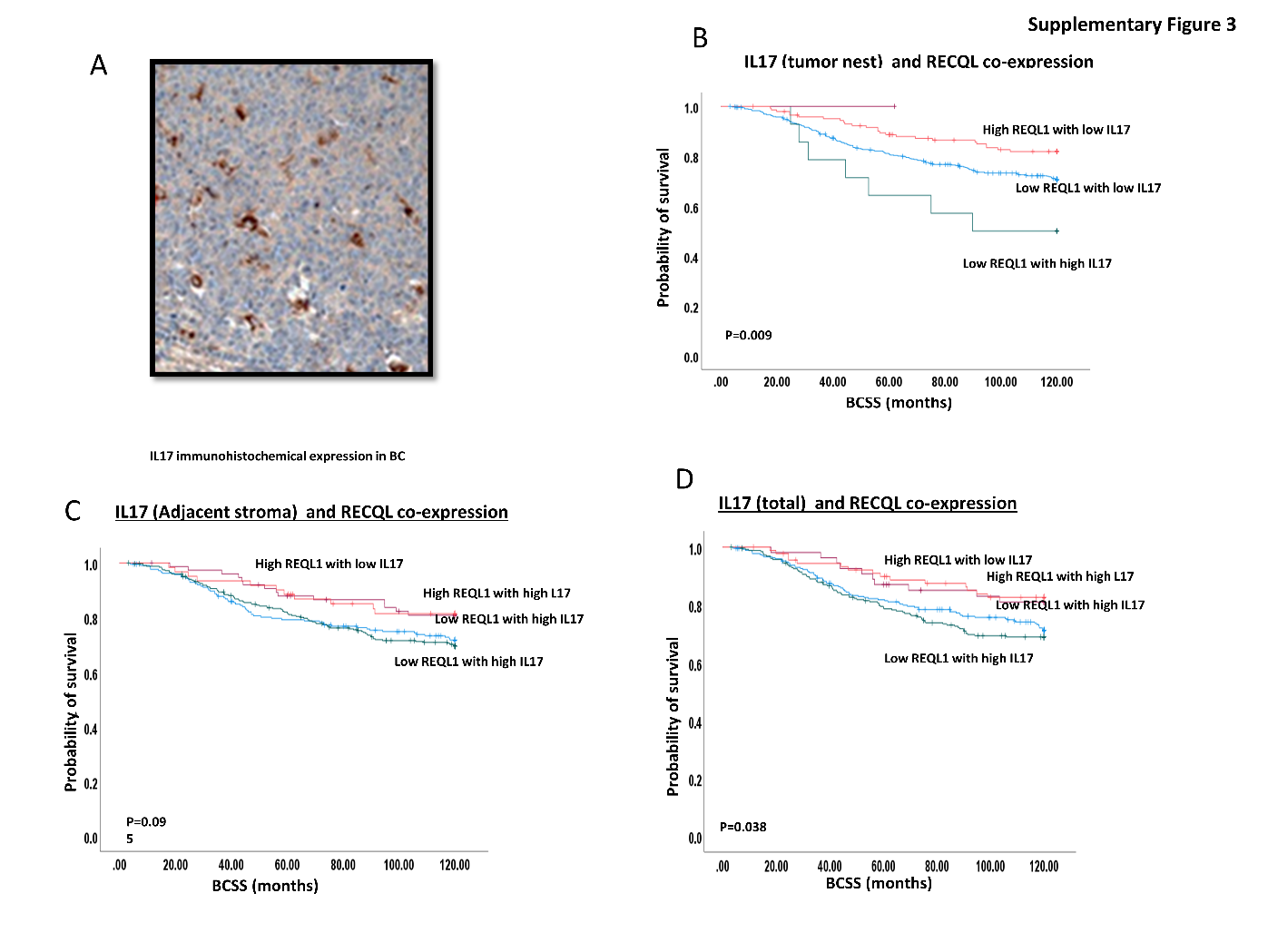


**Supplementary Figure 3. A.** Figure shows immunohistochemical expression of IL17 in breast cancer. **B.** Kaplan Meir curve shows the association between IL17 (tumour cells) and RECQL co-expression with BCSS. **C.** Kaplan Meir curve shows the association between IL17 (adjacent stroma) and RECQL co-expression with BCSS. **D.** Kaplan Meir curve shows the association between high IL17 total (within tumour and adjacent stroma) and RECQL co-expression with BCSS.

**
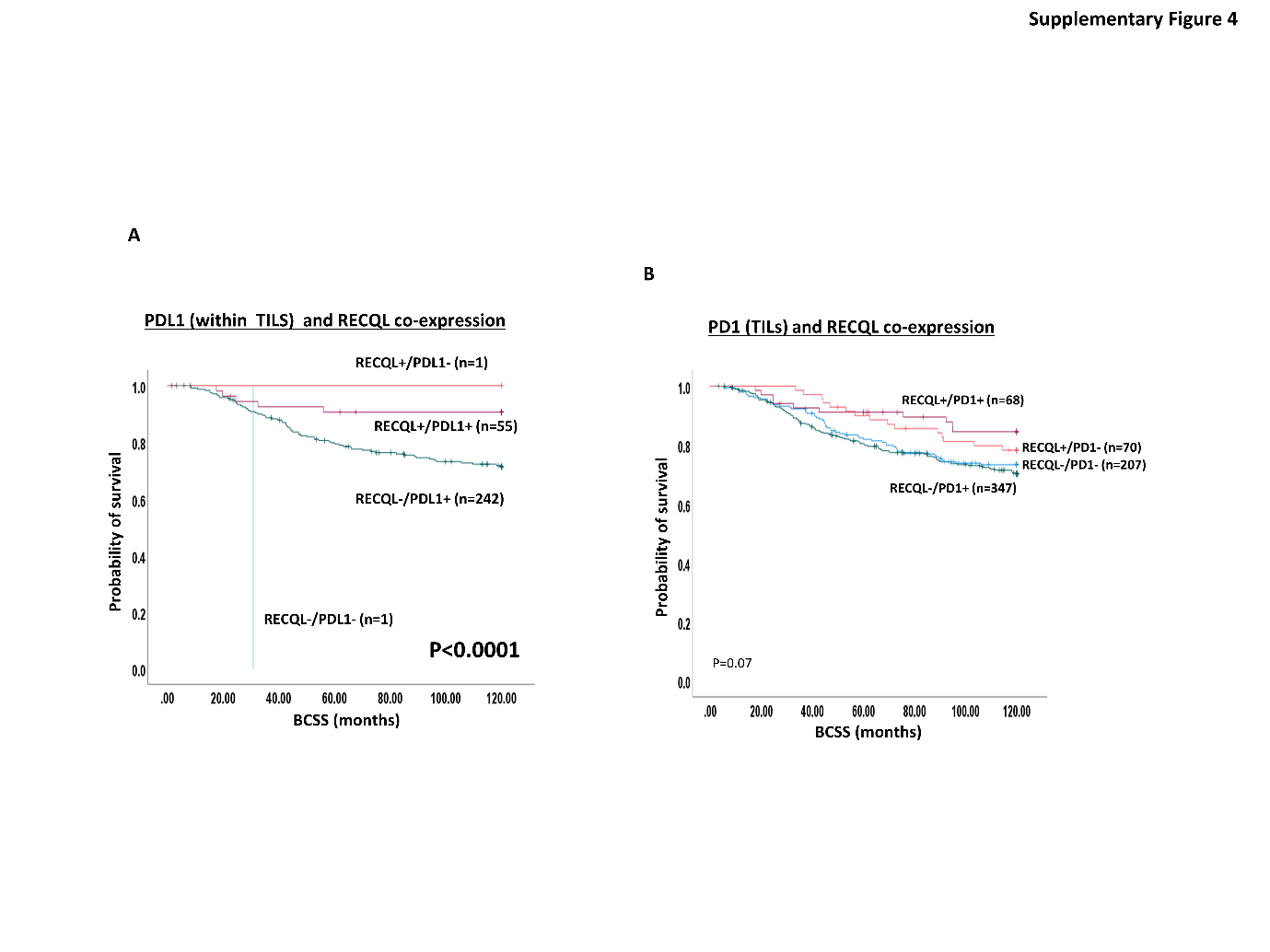
**

**Supplementary Figure 4:** Kaplan Meir curve shows the association between PDL1 positive (TILS) and RECQL co-expression with BCSS. **B.** Kaplan Meier curve showing prognostic significance of intra-tumoral PD1+TILs in RECQL deficient or proficient tumors.


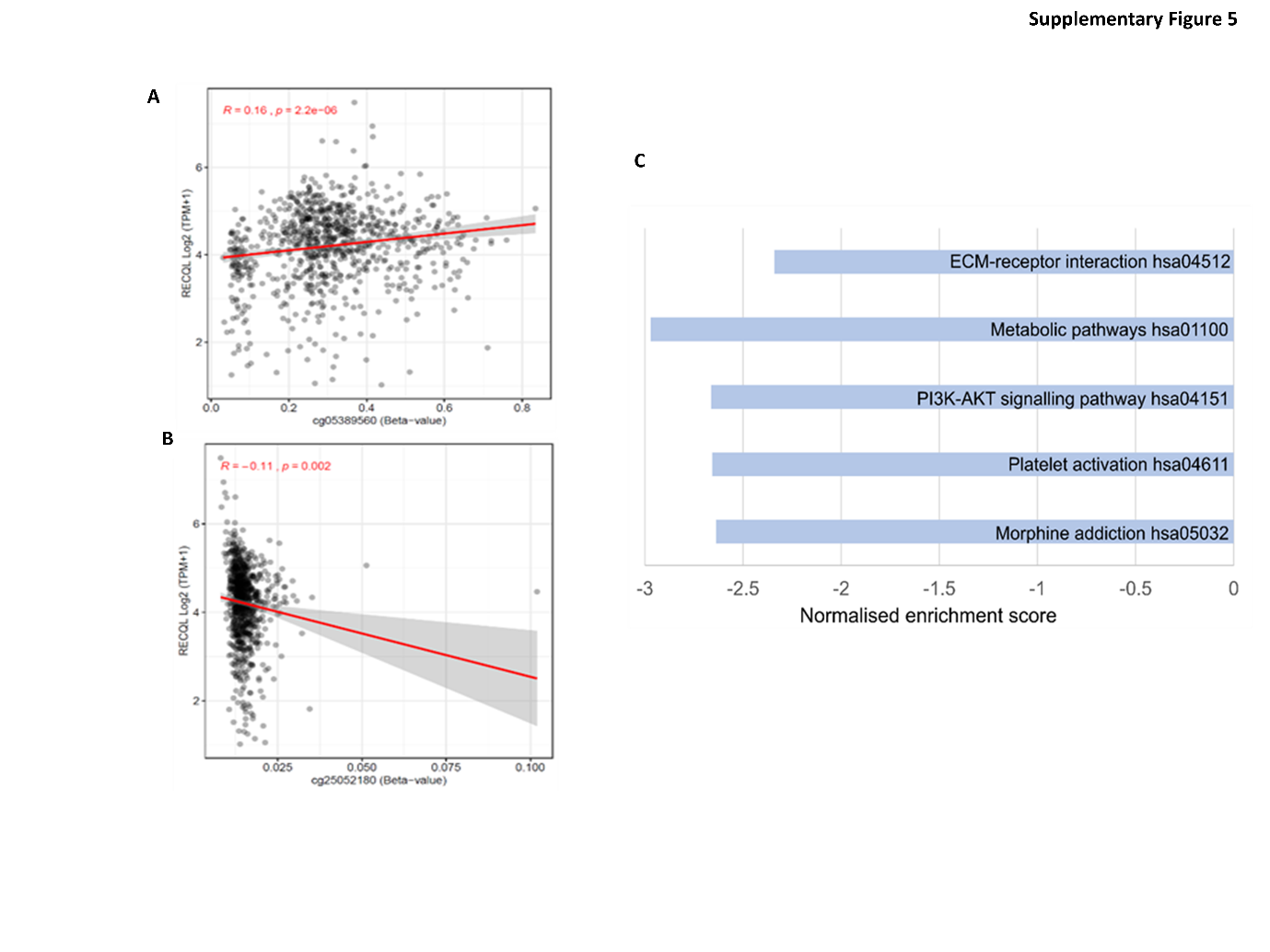


**Supplementary Figure 5:** HumanMethylation450K and expression data were from UCSC Xena tools. The *RECQ*L beta values were correlated with mRNA expression. Correlations shown are for CpG within CpG island in transcription start site *RECQL*. Probe cg5389560 represents the last gene internal CpG of the CpG island (**A**) and cg25052180 (**B**) represents the majority of CpG values within the CpG island having low DNA methylation <0.2 beta value. **C.** Top five GSEA pathways identified for the total differentially expressed genes between RECQL low tumours and RECQL high tumours from the TCGA RNA seq analysis.
