# Supplementary material for: Immune infiltration, aggressive pathology, and poor survival outcomes in RECQL helicase deficient breast cancers
Source: Neoplasia. 2023 Dec 21;47:100957. doi: 10.1016/j.neo.2023.100957 (PMC10777014; doi:10.1016/j.neo.2023.100957)
Supplement: Supplementary file 3 [file mmc3.docx]

**Supplementary Table 1**: Patient demographics in DCIS cohort.

| Clinicopathological parameter | Groups | Number of patients & (%) |
| --- | --- | --- |
| Age_50 | ≤50 | 115 (26%) |
|  | >50 | 334 (74%) |
| Extent | Localised (DCIS is in one quadrant) | 308 (69%) |
|  | Diffuse (DCIS is in more than one quadrant | 59 (31%) |
| Size_ VNPI | <16mm | 132 (29%) |
|  | 16-40mm | 183 (41%) |
|  | >40mm | 131 (30%) |
| Nuclear Grade | Low | 63 (14%) |
|  | Intermediate | 118 (26%) |
|  | High | 268 (60%) |
| Associated with Comedo Necrosis | No | 155 (66%) |
|  | Yes | 294 (34%) |
| Histological Pattern type | Single pattern | 214 (48%) |
|  | Mixture pattern | 223 (51%) |
| Associated with LCIS | No | 418 (93%) |
|  | Yes | 31 (7%) |
| Associated with Paget’s | No | 225 (8%) |
|  | Yes | 20 (92%) |
| Final Operation | BCS | 210 (47%) |
|  | Mastectomy | 238 (53%) |
| 2mm margin reference | Tumor on ink | 134 (30%) |
|  | Less than 2mm | 28 (6%) |
|  | 2mm and more | 268 (64%) |
| Menopausal status | Premenopausal | 107 (24%) |
|  | Postmenopausal | 342 (76%) |
| Estrogen status | Negative | 108 (28%) |
|  | Positive | 280 (78%) |
| Progesterone status | Negative | 172 (44%) |
|  | Positive | 216 (56%) |
| Her2 status | Negative | 304 (76%) |
|  | Positive | 98 (24%) |
| Proliferation index (Ki 67) | Low proliferation | 269 (76%) |
|  | High proliferation | 87 (24%) |

**Supplementary Table 2**: Clinicopathological associations between RECQL expression and pure DCIS.

| Parameters | Low RECQL.  No. (%) | High RECQL.  No. (%) | Total  No. (%) | (χ^2^ ) p-value |
| --- | --- | --- | --- | --- |
| Frequencies  Pure DCIS | 89 (20.0) | 360 (80.0) | 449 |  |
| Age  ≤50  >50 | 25 (22.0)  64 (19.0) | 90 (78.0)  270(81.0) | 115 (26.0)  334 (74.0) | 0.358  0.550 |
| Size*  <16mm  16-40mm  >40mm | 25 (20.0)  35 (19.0)  28 (21.0) | 107 (81.0)  148 (81.0)  103 (79.0) | 132 (30.0)  183 (41.0)  131 (29.0) | 0.318  0.853 |
| Extent  Localised  Diffuse | 57 (19.0)  13 (22.0) | 251 (81.0)  46 (78.0) | 308 (84.0)  59 (36.0) | 0.399  0.528 |
| Menopausal Status  Premenopausal  Postmenopausal | 25 (23.0)  64 (19.0) | 82 (39.0)  278 (44.0) | 107 (24.0)  342(76.0) | 1.109  0.292 |
| 2mm Margin  Tumor on ink  Less than 2mm  2mm and more | 33 (25.0)  7 (25.0)  17 (16.0) | 101 (75.0)  21 (75.0)  92 (84.0) | 134 (55.0)  28 (10.0)  109 (35.0) | 3.247  0.197 |
| Nuclear Grade  Low  Moderate  High | 10 (16.0)  15 (15.0)  64 (14.0) | 53 (84.0)  103 (87.0)  204 (76.0) | 63 (14.0)  118 (26.0)  268 (60.0) | 7.149  **0.028** |
| Comedo Necrosis  No  Yes | 30 (19.0)  59 (20.0) | 125(81.0)  235 (80.0) | 155 (35.0)  294 (65.0) | 0.032  0.857 |
| Type Mixture  Single Pattern  Mixed Pattern | 42 (20.0)  20(59.0) | 96 (50.0)  91 (41.0) | 214 (48.0)  223 (52.0) | 0.021  0.884 |
| LCIS  No  Yes | 87 (21.0)  2 (7.0) | 331 (79.0)  31 (93.0) | 418 (93.0)  31 (7.0) | 3.745  0.053 |
| Paget’s disease  No  Yes | 44 (20.0)  5 (25.0) | 181 (80.0)  15 (75.0) | 225 (92.0)  20 (8.0) | 0.340  0.560 |
| Final operation  BCS  Mastectomy | 43 (21.0)  46 (19.0) | 176 (79.0)  192(81.0) | 210 (47.0)  238 (53.0) | 0.092  0.761 |
| Radiotherapy  No  Yes | 73 (20.0)  16 (22.0) | 302 (80.0)  74 (78.0) | 375 (84.0)  74 (16.0) | 0.181  0.671 |
| Treatment Group  Mastectomy  BCS  BCS+RT | 46 (19.0)  28 (20.0)  15 (21.0) | 192 (81.0)  111 (80.0)  56 (79.0) | 283 (53.0)  139 (31.0)  71 (16.0) | 0.121  0.941 |
| Estrogen Status  Negative  Positive | 29 (27.0)  54 (19.0) | 79 (73.0)  226(81.0) | 108 (28.0)  280 (72.0) | 2.653  0.103 |
| Progesterone Status  Negative  Positive | 39 (23.0)  42 (19.0) | 133 (77.0)  174 (81.0) | 172 (44.0)  216 (56.0) | 0.605  0.437 |
| Her2 Status  Negative  Positive | 57 (19.0)  22 (22.0) | 247(81.0)  76 (78.0) | 304(76.0)  98 (24.0) | 0.642  0.423 |
| Proliferation index (Ki 67)  Low (<14%)  High (≥14%) | 59 (22.0)  12 (14.0) | 210 (78.0)  7 (75.0) | 269 (76.0)  87 (24.0) | 2.728  0.099 |

Significant *p* values are in **bold,** No: Number, X^2^: Chi square**.** RECQL1; ATP-dependent DNA helicase Q1, DCIS; Ductal Carcinoma *in Situ*, HER2; Human epidermal growth factor receptor 2. LCIS: Lobular Carcinoma *in Situ*, BCS: Breast conserving. * Size: based on Van Nuys Prognostic Index (VNPI).

**Supplementary Table 3**: Clinicopathological associations between RECQL expression and DCIS in mixed tumors.

| Parameters | Low RECQL.  No. (%) | High RECQL.  No. (%) | Total  No. (%) | (χ^2^ )  P value |
| --- | --- | --- | --- | --- |
| Frequencies  DCIS component existing with IBC | 87 (57.0) | 65(43.0) | 152 |  |
| Age  ≤50  >50 | 44 (60.0)  43 (54.0) | 29 (40.0)  36 (46.0) | 73 (48.0)  79 (52.0) | 0.529  0.467 |
| Size*  <16mm  16-40mm  >40mm | 28 (44.0)  51 (64.0)  8 (89.0) | 35 (56.0)  29 (36.0)  1 (11.0) | 63 (41.0)  80 (53.0)  9 (6.0) | 9.282  **0.010** |
| Nuclear Grade  Low  Moderate  High | 1 (17.0)  17 (49.0)  69 (62.0) | 5 (83.0)  18 (51.0)  42 (38.0) | 6 (4.0)  35 (23.0) 111(73.0) | 6.209  **0.045** |
| Comedo Necrosis  No  Yes | 18 (49.0)  69 (60.0) | 19 (51.0)  46 (40.0) | 37 (24.0)  115(76.0) | 1.471  0.225 |
| Radiotherapy  No  Yes | 19 (59.0)  67 (56.0) | 13 (41.0) 52 (44.0) | 32 (22.0)  119(78.0) | 0.097  0.755 |
| ER Status  Negative  Positive | 15 (68.0)  72 (55.0) | 7 (32.0)  58 (45.0) | 22 (14.0)  130(86.0) | 1.259  0.262 |
| Extent  Localised  Diffuse | 26 (55.0)  61 (58.0) | 21 (45.0)  44 (42.0) | 47 (31.0)  105(69.0) | 0.102  0.749 |
| DCIS Types  Cribriform  Solid  Pap. & micropapillary  Crib & mixed  Solid & mixed | 7 (47.0)  41 (61.0)  2 (67.0)  5 (71.0)  32 (53.0) | 8 (53.0)  26 (39.0)  1 (33.0)  2 (29.0)  28(47.0) | 15 (10.0)  67 (44.0)  3 (2.0)  7 (5.0)  60 (39.0) | 2.172  0.704 |
| Final Operation  Mastectomy  BCS | 46 (66.0)  41(50.0) | 24(34.0)  41(50.0) | 70 (46.0)  82 (54.0) | 3.810  0.051 |
| Stage  Stage 1  Stage 2  Stage 3 | 49 (56.0)  24 (53.0)  14 (70.0) | 38 (34.0)  21 (47.0)  6 (30.0) | 87 (57.0)  45 (30.0)  20 (13.0) | 1.641  0.440 |
| L.N Metastasis  No  Yes | 49 (56.0)  38 (58.0) | 38 (44.0)  27 (42.0) | 87 (57.0)  65 (43.0) | 0.070  0.792 |

Significant *p* values are in **bold,** No: Number, X^2^: Chi square**.** RECQL1: ATP-dependent DNA helicase Q1,

DCIS; Ductal Carcinoma *in Situ*, BCS: Breast conserving. * Size: based on Van Nuys Prognostic Index (VNPI).

**Supplementary Table** **4:** Multivariate analysis in DCIS.

| **All recurrences** | | | | |
| --- | --- | --- | --- | --- |
| Parameters | p-value | HR | 95% confidence interval (CI) | |
|  |  |  | Lower | Upper |
| RECQL | **0.028** | 0.538 | 0.309 | 0.936 |
| Age | **0.004** | 0.455 | 0.265 | 0.780 |
| DCIS size | **0.001** | 0.398 | 0.228 | 0.695 |
| **IBC only** | | | | |
| Parameters | p-value | HR | 95% confidence interval (CI) | |
|  |  |  | Lower | Upper |
| RECQL | **0.029** | 2.913 | 1.115 | 7.608 |
| Age | 0.105 | 0444 | 0.166 | 1.186 |
| DCIS size | 0.577 | 1.252 | 0.569 | 2.754 |
| Nuclear grade | 0.944 | 0.981 | 0.571 | 1.685 |

**Supplementary Table 5:** Patient demographics in invasive breast cancer cohort.

| Variables | No (%) |
| --- | --- |
| **Tumor size**  ≤ 2cm  > 2cm | 358 (49%)  368 (51%) |
| **Grade** 1  Grade 2  Grade 3 | 98 (13%)  252 (35%)  376 (52%) |
| **Tubule formation**  1  2  3 | 32 (4%)  240 (33%)  454 (62%) |
| **Polymorphism**  1  2  3 | 23 (3%)  254 (35%)  449 (62%) |
| **Mitosis**  1  2  3 | 241 (33%)  145 (20%)  340 (47%) |
| **Histologic tumor types**  No Special Type (NST)  Lobular  Other special types  NST mixed | 462 (64%)  74 (10%)  22 (3%)  168 (23%) |
| **Lymhovascular invasion**  Absent  Present | 469 (65%)  257 (35%) |
| **Lymph node status**  Absent  Present | 446 (61%)  280 (39%) |
| **Nottingham prognostic index**  Good prognostic group  Moderate prognostic group  Poor prognostic group | 204 (28%)  406 (56%)  116 (16%) |
| **ER status**  Negative  Positive | 203 (28%)  521 (72%) |
| **PR status**  Negative  Positive | 304 (42%)  409 (58%) |
| **HER2 status**  Negative  Positive | 618 (85%)  102 (15%) |
| **Ki67 expression**  Low ≤ 14%  High >14% | 239 (33%)  341 (47%) |
| **Molecular subtypes**  Luminal A  Luminal B  HER2 enriched  Triple negative | 203 (33%)  219 (36%)  55 (9%)  134 (22%) |

* Number of cases for which data were available. NPI; Nottingham prognostic index, PG; prognostic group

**Supplementary Table 6:** Antigens, primary antibodies, clone, source, optimal dilution and scoring system used for each immunohistochemical marker.

| **Antigen** | **Antibody** | **Clone** | **Source** | **Antigen Retrieval** | **Dilution / Incubation Time** | **Distribution** | **Scoring**  **system** | **Cut-offs** |
| --- | --- | --- | --- | --- | --- | --- | --- | --- |
| BRCA1 | BRCA1 | MS110 | Calbiochem | Citrate pH6 | 1:100  60 min | Nuclear | % of positive cells | <25% (negative) |
| ATM | Rabbit MAb anti-ATM | Y170 | Abcam | Citrate pH6 | 1:100  18 hours | Nuclear | % of positive cells | <25% (negative) |
| Pol β | Rabbit anti-polβ | Ab26343 | Abcam | Citrate pH6 | 1:200  60 min | Nuclear | H- Score | ≥100  (Median H-score, positive) |
| BLM | Rabbit anti BLM | Polyclonal | Novus-Biologicals | Citrate pH6 | 1:100  18 Hours | Nuclear | H- Score | ≥50  (Median H-score, positive) |
| RECQL4 | Rabbit Anti  RECQL4 | Polyclonal | Novus Biologicals | Citrate pH6 | 1:1000  60 min | Nuclear | H-score | Nuclear ≥215 (Median H-score High) |
| CHK2 | Rabbit Anti  CHK2 | Polyclonal | Abcam | Citrate pH6 | 1:100  60 min | Nuclear | H- Score | ≥100  (Median H-score, positive) |
| PARP1 | Mouse MAb Anti-PARP1 | 7D3-6 | BD pharmingen | Citrate pH6 | 1:1000 | Nuclear | % of positive cells | ≥10% (positive) |
| TOP2A | Mouse MAb | KiS1 | Dako-Cytomation | Citrate pH6 | 1:150  60 min | Nuclear | % of positive cells | >25% (positive) |
| DNA-PKcs | Mouse MAb Anti- | 3H6 | Abcam | Citrate pH6 | 1:1000  20 min | Nuclear | H-score | >260 ((Mean H-score, positive) |
| RAD50 | Mouse anti-RAD50 | Polyclonal | Abcam | Citrate pH6 | 1:70  20 min | Nuclear | H-score | >40 |
| NBS1 | Rabbit Anti-NBS1 | N3162 | Sigma | Citrate pH6 | 1:100  18hours | Nuclear cytoplasmic | H-score | Low nuclear, Median H score <90  Low cytoplasmic, Median H-score <70 |
| MRE11 | Mouse MAb Anti-MRE11 | ab214 | Abcam | Citrate pH6 | 1:800  18hours | Nuclear cytoplasmic | H-score | Low nuclear, Median H-score  <90  Low cytoplasmic, Median H-score <10 |
| ERCC1 | Mouse anti-ERCC1 | 4F9 | Dako Ltd | citrate pH 6.0 | 1:150  30 min | Nuclear | H-score | ≥ 130 |
| RPA1 | Rabbit Anti-RPA70 | ab79398 | Abcam | Citrate pH6 | 1:100 , 1h room temperature | Nuclear | H-score | 100 |
| RPA2 | Mouse Anti-RPA32 | ab2175 | Abcam | Citrate pH6 | 1:100 , 1h room temperature | Nuclear | H-score | 70 |
| RPA3 | Rabbit Anti-RPA14 | ab97436 | Abcam | Citrate pH6 | 1:50, 1h room temperature | Nuclear  cytoplasmic | H-score | Nuclear 50- Cyto 40 |
| RECQL5 | Rabbit anti RecqL5 | Polyclonal | SigmaAldrich | Citrate pH6 | 1:100  60 min | Nuclear | H - Score | ≥10 (positive) |
| ATR | Mouse MAb Anti-ATR | 1E9 | Novus Biologicals | Citrate pH6 | 1:20  18 hours | Nuclear | H-score | ≥60 (High) |
| pCHK1 | Rabbit anti-pChk1 | Ab58567 | Abcam | Citrate pH6 | 1:140  60 min | Nuclear | H-score | ≥50 (High) |
| RAD51 | Mouse MAb Anti-RAD50 | ab89 | Abcam | Citrate pH6 | 1:100  18hours | Nuclear | H-score | Low nuclear, Median H-score  <100 |
| ER | Mouse MAb anti-ER-α | SP1 | Dako-Cytomation | Citrate pH6 | 1:150  30 min | Nuclear | Allred score | ≥3 (positive) |
| ER | Mouse MAb anti-ER-α | EP1 | Dako-Cytomation | Citrate pH6 | 1:80  30 min | Nuclear | % positive cells | ≥1% positive |
| PR | Mouse MAb anti-PR | PgR636 | Dako-Cytomation | Citrate pH6 | 1:125  30 min | Nuclear | % positive cells | ≥1% positive |
| HER2 | Rabbit antihuman c-erbB2 | polyclonal | Dako-Cytomation | None | 1:400  60 min | Membrane | See text | See text |
| CD8 | Mouse MAb Anti-CD8 | 1A5 | Vector Laboratories | Citrate pH6 | 1:50  20 min | Membrane | See text | See text |
| FOXP3 | Mouse MAb Anti-FOXP3 | 236A/E7 | Abcam | Citrate pH6 | 1:100  60 min | Stroma | positive cell counts | ≥3 positive |
| PD-1 | Mouse MAb Anti-PD-1 | EH33 | Cell Signalling Technology | Citrate pH6 | 1:75  24 hours | Stroma | % positive cells | ≥5% positive |
| PD-L1 | Rabbit MAb Anti-PD-L1 | E1L3N | Cell Signalling Technology | Epitope retrieval solution 2, pH9, 95°C, 45 min | 1:25  24 hours | Membrane  Cytoplasm  Stroma | % positive in tumour  % positive cells in stroma | ≥1% positive |
| IL17 | Goat polyclonal anti-human IL-17 | polyclonal | Santa Cruz Biotechnology | Citrate pH6 | 1:100  60 min | cytoplasmic and the nuclei | 0 for lack of inflammation, 1+ for mild inflammation and 2+ for marked inflammation. | ≥1 positive |

All sections were pre-treated with microwave antigen retrieval using 0.1% citrate buffer (pH 6) except for HER2 (no pre-treatment).

| DNA repair marker | Correlation coefficient | P value | Number of samples |
| --- | --- | --- | --- |
| RPA1 | 0.34 | <0.0001 | 467 |
| RPA2 | 0.12 | 0.064 | 257 |
| KU70 | 0.16 | <0.0001 | 582 |
| MRE11 | 0.41 | <0.0001 | 457 |
| RAD50 | 0.27 | <0.0001 | 295 |
| RAD51 | -0.002 | 0.96 | 528 |
| BRCA1 | 0.2 | <0.0001 | 724 |
| BRCA2 | 0.076 | 0.066 | 588 |
| ATM | 0.11 | 0.012 | 547 |
| ATR | 0.026 | 0.48 | 755 |
| XRCC1 | 0.33 | <0.0001 | 683 |
| PolyB | 0.23 | <0.0001 | 798 |
| NBS1 | 0.13 | 0.001 | 668 |
| pChk1 | 0.22 | <0.0001 | 898 |
| CHK2 | 0.26 | <0.0001 | 565 |
| DNA-PKcs | 0.2 | <0.0001 | 483 |
| RECQL4 | 0.19 | <0.0001 | 712 |
| RECQL5 | 0.18 | <0.0001 | 755 |
| BLM | 0.24 | <0.0001 | 752 |
| ERCC1 | 0.26 | <0.0001 | 695 |
| PARP1_Cleaved | 0.27 | <0.0001 | 664 |
| PARP1_non-cleaved | 0.17 | <0.0001 | 714 |

**Supplementary Table 7.** RECQL and correlation to other DNA repair markers in breast cancer.

**Supplementary Table 8.** Clinicopathological significance of RECQL and CD8+ (within adjacent stroma) co-expression in breast cancers.

|  | **RECQL-/CD8-** | **RECQL+/CD8+** | **RECQL+/CD8-** | **RECQL-/CD8+** | **X^2^** |
| --- | --- | --- | --- | --- | --- |
|  |  |  |  |  | ***P*-value** |
| **Tumor size** | | | | | |
| ≤ 2cm | 152 (53%) | 26 (45%) | 55 (58%) | 99 (40%) | 13.79  **0.003** |
| > 2cm | 136 (47%) | 32 (55%) | 39 (42%) | 150 (60%) |  |
| **Tumor grade** | | | | | |
| Grade 1 | 44 (15%) | 9 (16%) | 19 (20%) | 17 (7%) | 51.524  **<0.0001** |
| Grade2 | 113 (39%) | 14 (24%) | 45 (48%) | 61 (24%) |  |
| Grade 3 | 131 (46%) | 35 (60%) | 30 (32%) | 171 (69%) |  |
| **Tubule formation** | | | | | |
| 1 | 15 (5%) | 4 (7%) | 7 (7%) | 7 (3%) | 14.334  **0.026** |
| 2 | 105 (37%) | 16 (28%) | 34 (36%) | 64 (26%) |  |
| 3 | 168 (58%) | 38 (65%) | 53 (57%) | 178 (71%) |  |
| **Pleomorphism** | | | | | |
| 1 | 11 (4%) | 1 (2%) | 2 (2%) | 6 (3%) | 50.436  **<0.0001** |
| 2 | 114 (40%) | 18 (31%) | 52 (55%) | 48 (19%) |  |
| 3 | 163 (56%) | 39 (67%) | 40 (43%) | 195 (78%) |  |
| **Mitosis** | | | | | |
| 1 | 107 (37%) | 19 (33%) | 47 (50%) | 49 (20%) | 55.523  **<0.0001** |
| 2 | 69 (24%) | 6 (10%) | 19 (20%) | 41 (16%) |  |
| 3 | 112 (39%) | 33 (57%) | 28 (30%) | 159 (64%) |  |
| **Histologic types** | | | | | |
| No special type (NST) | 167 (58%) | 40 (69%) | 45 (48%) | 195 (78%) | 41.793  **<0.0001** |
| Lobular | 38 (13%) | 2 (3%) | 15 (16%) | 13 (5%) |  |
| Other special types | 10 (4%) | 2 (3%) | 4 (4%) | 6 (3%) |  |
| Mixed NST | 73 (25%) | 14 (25%) | 30 (32%) | 35 (14%) |  |
| **Lymph node status** | | | | | |
| Absent | 172 (60%) | 38 (66%) | 67 (71%) | 145 (58%) | 5.665  0.129 |
| Present | 116 (40%) | 20 (34%) | 27 (29%) | 104 (42%) |  |
| **Lymphovascular invasion** | | | | | |
| Absent | 199 (69%) | 36 (62%) | 66 (70%) | 141 (57%) | 10.805  **0.013** |
| Present | 89 (31%) | 22 (38%) | 28 (30%) | 108 (43%) |  |
| **Nottingham Prognostic index** | | | | | |
| Good prognostic group | 90 (31%) | 16 (27%) | 44 (47%) | 39 (16%) | 43.588  **<0.0001** |
| Moderate prognostic group | 156 (54%) | 30 (52%) | 45 (48%) | 154 (62%) |  |
| Poor prognostic group | 42 (15%) | 12 (21%) | 5 (5%) | 56 (22%) |  |
| **ER status** | | | | | |
| Negative | 62 (22%) | 20 (34%) | 17 (18%) | 96 (39%) | 25.15  **<0.0001** |
| Positive | 225 (78%) | 38 (66%) | 77 (82%) | 153 (61%) |  |
| **PR status** | | | | | |
| Negative | 116 (41%) | 23 (42%) | 28 (30%) | 128 (52%) | 14.535  **0.002** |
| Positive | 167 (59%) | 32 (58%) | 65 (70%) | 119 (48%) |  |
| **Ki67 expression** | | | | | |
| Low≤14 | 109 (46%) | 16 (32%) | 47 (60%) | 52 (27%) | 31.308  **<0.0001** |
| High>14 | 129 (54%) | 34 (68%) | 31 (40%) | 140 (73%) |  |
| **Molecular subtypes** | | | | | |
| Luminal A | 96 (38%) | 14 (28%) | 44 (55%) | 38 (19%) | 51.438  **<0.0001** |
| Luminal B | 98 (39%) | 19 (38%) | 20 (25%) | 78 (38%) |  |
| Triple negative | 40 (16%) | 11 (22%) | 14 (18%) | 62 (30%) |  |
| HER2 + | 18 (7%) | 6 (12%) | 2 (2%) | 27 (13%) |  |

**Supplementary Table 9.** Clinicopathological significance of RECQL and CD8+ (within distant stroma) co-expression in breast cancers.

|  | **RECQL-/CD8-** | **RECQL+/CD8+** | **RECQL+/CD8-** | **RECQL-/CD8+** | **X^2^** |
| --- | --- | --- | --- | --- | --- |
|  |  |  |  |  | ***P*-value** |
| **Tumor size** | | | | | |
| ≤ 2cm | 60 (45%) | 68 (57%) | 13 (39%) | 191 (47%) | 5.571  0.134 |
| > 2cm | 74 (55%) | 51 (43%) | 20 (61%) | 212 (53%) |  |
| **Tumor grade** | | | | | |
| Grade 1 | 16 (12%) | 24 (20%) | 4 (12%) | 45 (11%) | 13.69  **0.033** |
| Grade2 | 39 (29%) | 48 (40%) | 11 (33%) | 135 (34%) |  |
| Grade 3 | 79 (59%) | 47 (40%) | 18 (55%) | 223 (55%) |  |
| **Tubule formation** | | | | | |
| 1 | 7 (5%) | 8 (7%) | 3 (9%) | 15 (4%) | 4.385  0.625 |
| 2 | 39 (29%) | 38 (32%) | 12 (36%) | 130 (32%) |  |
| 3 | 88 (66%) | 73 (61%) | 18 (55%) | 258 (64%) |  |
| **Pleomorphism** | | | | | |
| 1 | 6 (5%) | 3 (3%) | 0 (0%) | 11 (3%) | 15.735  **0.015** |
| 2 | 42 (31%) | 56 (47%) | 14 (42%) | 120 (30%) |  |
| 3 | 86 (64%) | 60 (50%) | 19 (58%) | 272 (67%) |  |
| **Mitosis** | | | | | |
| 1 | 32 (24%) | 59 (50%) | 7 (21%) | 124 (31%) | 23.007  **0.001** |
| 2 | 29 (22%) | 18 (15%) | 7 (21%) | 81 (20%) |  |
| 3 | 73 (54%) | 42 (35%) | 19 (58%) | 198 (49%) |  |
| **Histologic types** | | | | | |
| No special type (NST) | 94 (70%) | 62 (52%) | 23 (70%) | 268 (67%) | 15.994  0.067 |
| Lobular | 9 (7%) | 17 (14%) | 0 (0%) | 42 (10%) |  |
| Other special types | 3 (2%) | 5 (4%) | 1 (3%) | 13 (3%) |  |
| Mixed NST | 28 (21%) | 35 (30%) | 9 (27%) | 80 (20%) |  |
| **Lymph node status** | | | | | |
| Absent | 82 (61%) | 83 (70%) | 22 (67%) | 235 (58%) | 5.494  0.139 |
| Present | 52 (39%) | 36 (30%) | 11 (33%) | 168 (42%) |  |
| **Lymphovascular invasion** | | | | | |
| Absent | 86 (64%) | 80 (67%) | 22 (67%) | 254 (63%) | 0.802  0.849 |
| Present | 48 (36%) | 39 (33%) | 11 (33%) | 149 (37%) |  |
| **Nottingham Prognostic index** | | | | | |
| Good prognostic group | 35 (26%) | 53 (45%) | 7 (21%) | 94 (23%) | 22.912  **0.001** |
| Moderate prognostic group | 74 (55%) | 54 (45%) | 21 (64%) | 236 (59%) |  |
| Poor prognostic group | 25 (19%) | 12 (10%) | 5 (15%) | 73 (18%) |  |
| **ER status** | | | | | |
| Negative | 44 (33%) | 29 (24%) | 8 (24%) | 114 (28%) | 2.53  0.47 |
| Positive | 90 (67%) | 90 (76%) | 25 (76%) | 288 (72%) |  |
| **PR status** | | | | | |
| Negative | 65 (49%) | 37 (32%) | 14 (42%) | 179 (45%) | 7.987  **0.046** |
| Positive | 68 (51%) | 78 (68%) | 19 (58%) | 218 (55%) |  |
| **Ki67 expression** | | | | | |
| Low≤14 | 43 (40%) | 53 (54%) | 10 (35%) | 118 (37%) | 9.42  **0.024** |
| High>14 | 65 (60%) | 46 (46%) | 19 (65%) | 204 (63%) |  |
| **Molecular subtypes** | | | | | |
| Luminal A | 34 (29%) | 50 (51%) | 8 (25%) | 100 (29%) | 25.539  **0.002** |
| Luminal B | 38 (33%) | 23 (24%) | 16 (50%) | 138 (41%) |  |
| Triple negative | 33 (28%) | 18 (18%) | 7 (22%) | 69 (20%) |  |
| HER2 + | 11 (10%) | 7 (7%) | 1 (3%) | 34 (10%) |  |

**Supplementary Table 10.** Clinicopathological significance of RECQL and total CD8+ (within tumor cell nest, adjacent and distant stroma) co-expression in breast cancers.

|  | **RECQL-/CD8-** | **RECQL+/CD8+** | **RECQL+/CD8-** | **RECQL-/CD8+** | **X^2^** |
| --- | --- | --- | --- | --- | --- |
|  |  |  |  |  | ***P*-value** |
| **Tumor size** | | | | | |
| ≤ 2cm | 35 (53%) | 69 (53%) | 11 (52%) | 217 (46%) | 3.231  0.357 |
| > 2cm | 31 (47%) | 60 (47%) | 10 (48%) | 256 (54%) |  |
| **Tumor grade** | | | | | |
| Grade 1 | 12 (18%) | 24 (19%) | 4 (19%) | 49 (10%) | 15.981  **0.014** |
| Grade2 | 24 (36%) | 48 (37%) | 10 (48%) | 151 (32%) |  |
| Grade 3 | 30 (46%) | 57 (44%) | 7 (33%) | 273 (58%) |  |
| **Tubule formation** | | | | | |
| 1 | 6 (9%) | 8 (6%) | 3 (14%) | 16 (3%) | 13.586  **0.035** |
| 2 | 22 (33%) | 39 (30%) | 10 (48%) | 148 (31%) |  |
| 3 | 38 (58%) | 82 (64%) | 8 (38%) | 309 (66%) |  |
| **Pleomorphism** | | | | | |
| 1 | 6 (9%) | 3 (2%) | 0 (0%) | 11 (2%) | 28.317  **<0.0001** |
| 2 | 28 (42%) | 58 (45%) | 10 (48%) | 136 (29%) |  |
| 3 | 32 (49%) | 68 (53%) | 11 (52%) | 326 (69%) |  |
| **Mitosis** | | | | | |
| 1 | 21 (32%) | 59 (46%) | 7 (33%) | 135 (29%) | 18.333  **0.005** |
| 2 | 18 (27%) | 19 (15%) | 6 (29%) | 92 (19%) |  |
| 3 | 27 (41%) | 51 (39%) | 8 (38%) | 246 (52%) |  |
| **Histologic types** | | | | | |
| No special type (NST) | 39 (59%) | 71 (55%) | 13 (62%) | 324 (69%) | 15.286  0.083 |
| Lobular | 6 (9%) | 17 (13%) | 0 (0%) | 45 (9%) |  |
| Other special types | 1 (2%) | 5 (4%) | 1 (5%) | 15 (3%) |  |
| Mixed NST | 20 (30%) | 36 (28%) | 7 (33%) | 89 (19%) |  |
| **Lymph node status** | | | | | |
| Absent | 45 (68%) | 88 (68%) | 16 (76%) | 273 (58%) | 8.437  **0.038** |
| Present | 21 (32%) | 41 (32%) | 5 (24%) | 200 (42%) |  |
| **Lymphovascular invasion** | | | | | |
| Absent | 48 (73%) | 84 (65%) | 16 (76%) | 294 (62%) | 4.305  0.230 |
| Present | 18 (27%) | 45 (35%) | 5 (24%) | 179 (38%) |  |
| **Nottingham Prognostic index** | | | | | |
| Good prognostic group | 24 (36%) | 53 (41%) | 7 (33%) | 105 (22%) | 26.955  **<0.0001** |
| Moderate prognostic group | 35 (53%) | 59 (46%) | 14 (67%) | 277 (59%) |  |
| Poor prognostic group | 7 (11%) | 17 (13%) | 0 (0%) | 91 (19%) |  |
| **ER status** | | | | | |
| Negative | 12 (18%) | 32 (25%) | 5 (24%) | 146 (31%) | 5.920  0.116 |
| Positive | 54 (82%) | 97 (75%) | 16 (76%) | 326 (69%) |  |
| **PR status** | | | | | |
| Negative | 25 (39%) | 42 (34%) | 7 (33%) | 221 (47%) | 9.316  **0.025** |
| Positive | 40 (61%) | 83 (66%) | 14 (67%) | 246 (53%) |  |
| **Ki67 expression** | | | | | |
| Low≤14 | 28 (52%) | 53 (49%) | 10 (56%) | 133 (35%) | 12.312  **0.006** |
| High>14 | 26 (48%) | 55 (51%) | 8 (44%) | 245 (65%) |  |
| **Molecular subtypes** | | | | | |
| Luminal A | 24 (43%) | 50 (46%) | 8 (40%) | 110 (27%) | 21.379  **0.011** |
| Luminal B | 20 (36%) | 30 (28%) | 7 (35%) | 158 (39%) |  |
| Triple negative | 11 (20%) | 21 (19%) | 4 (20%) | 91 (23%) |  |
| HER2 + | 1 (1%) | 7 (7%) | 1 (5%) | 44 (11%) |  |

**Table 11.** Clinicopathological significance of RECQL and FOXP3+ (within adjacent stroma) co-expression in breast cancers.

|  | **RECQL-/FOXP3-** | **RECQL+/FOXP3+** | **RECQL+/FOXP3-** | **RECQL-/FOXP3+** | **X^2^** |
| --- | --- | --- | --- | --- | --- |
|  |  |  |  |  | ***P*-value** |
| **Tumor size** | | | | | |
| ≤ 2cm | 223 (51%) | 8 (29%) | 81 (58%) | 46 (37%) | 17.35  **0.001** |
| > 2cm | 212 (49%) | 20 (71%) | 58 (42%) | 78 (63%) |  |
| **Tumor grade** | | | | | |
| Grade 1 | 62 (14%) | 2 (7%) | 31 (22%) | 3 (2%) | 72.785  **<0.0001** |
| Grade2 | 164 (38%) | 6 (21%) | 62 (45%) | 20 (16%) |  |
| Grade 3 | 209 (48%) | 20 (72%) | 46 (33%) | 101 (82%) |  |
| **Tubule formation** | | | | | |
| 1 | 21 (5%) | 1 (4%) | 10 (7%) | 0 (0%) | 28.833  **<0.0001** |
| 2 | 156 (36%) | 7 (25%) | 54 (39%) | 23 (18%) |  |
| 3 | 258 (59%) | 20 (71%) | 75 (54%) | 101 (82%) |  |
| **Pleomorphism** | | | | | |
| 1 | 18 (4%) | 0 (0%) | 4 (3%) | 1 (1%) | 51.006  **<0.0001** |
| 2 | 153 (35%) | 6 (21%) | 75 (54%) | 20 (16%) |  |
| 3 | 264 (61%) | 22 (79%) | 60 (43%) | 103 (83%) |  |
| **Mitosis** | | | | | |
| 1 | 157 (36%) | 6 (21%) | 71 (51%) | 7 (6%) | 82.932  **<0.0001** |
| 2 | 95 (22%) | 2 (7%) | 25 (18%) | 23 (18%) |  |
| 3 | 183 (42%) | 20 (72%) | 43 (31%) | 94 (76%) |  |
| **Histologic types** | | | | | |
| No special type (NST) | 262 (60%) | 25 (88%) | 63 (45%) | 112 (90%) | 70.563  **<0.0001** |
| Lobular | 49 (11%) | 1 (4%) | 22 (16%) | 2 (2%) |  |
| Other special types | 16 (4%) | 1 (4%) | 5 (4%) | 0 (0%) |  |
| Mixed NST | 108 (25%) | 1 (4%) | 49 (35%) | 10 (8%) |  |
| **Lymph node status** | | | | | |
| Absent | 251 (58%) | 17 (61%) | 98 (70%) | 80 (65%) | 7.887  **0.048** |
| Present | 184 (42%) | 11 (39%) | 41 (30%) | 44 (35%) |  |
| **Lymphovascular invasion** | | | | | |
| Absent | 271 (62%) | 15 (54%) | 104 (75%) | 79 (64%) | 8.888  **0.031** |
| Present | 164 (38%) | 13 (46%) | 35 (25%) | 45 (36%) |  |
| **Nottingham Prognostic index** | | | | | |
| Good prognostic group | 123 (28%) | 6 (21%) | 61 (44%) | 14 (11%) | 42.316  **<0.0001** |
| Moderate prognostic group | 245 (56%) | 14 (50%) | 67 (48%) | 80 (65%) |  |
| Poor prognostic group | 67 (16%) | 8 (29%) | 11 (8%) | 30 (24%) |  |
| **ER status** | | | | | |
| Negative | 101 (23%) | 11 (39%) | 25 (18%) | 66 (53%) | 52.364  **<0.0001** |
| Positive | 333 (77%) | 17 (61%) | 113 (82%) | 58 (47%) |  |
| **PR status** | | | | | |
| Negative | 172 (40%) | 12 (44%) | 41 (31%) | 79 (64%) | 31.542  **<0.0001** |
| Positive | 256 (60%) | 15 (56%) | 93 (69%) | 45 (36%) |  |
| **Ki67 expression** | | | | | |
| Low≤14 | 154 (44%) | 4 (18%) | 65 (57%) | 16 (17%) | 41.773  **<0.0001** |
| High>14 | 194 (56%) | 18 (82%) | 49 (43%) | 80 (83%) |  |
| **Molecular subtypes** | | | | | |
| Luminal A | 131 (36%) | 4 (18%) | 58 (50%) | 10 (9%) | 72.962  **<0.0001** |
| Luminal B | 140 (39%) | 9 (41%) | 35 (30%) | 35 (32%) |  |
| Triple negative | 62 (17%) | 8 (36%) | 17 (14%) | 47 (43%) |  |
| HER2 + | 29 (8%) | 1 (5%) | 7 (6%) | 18 (16%) |  |

**Supplementary Table 12.** Clinicopathological significance of RECQL and FOXP3+ (within distant stroma) co-expression in breast cancers.

|  | **RECQL-/FOXP3-** | **RECQL+/FOXP3+** | **RECQL+/FOXP3-** | **RECQL-/FOXP3+** | **X^2^** |
| --- | --- | --- | --- | --- | --- |
|  |  |  |  |  | ***P*-value** |
| **Tumor size** | | | | | |
| ≤ 2cm | 111 (46%) | 50 (56%) | 40 (51%) | 157 (49%) | 2.313  0.510 |
| > 2cm | 128 (54%) | 40 (44%) | 38 (49%) | 162 (51%) |  |
| **Tumor grade** | | | | | |
| Grade 1 | 35 (15%) | 16 (18%) | 17 (22%) | 30 (9%) | 53.317  **<0.0001** |
| Grade2 | 106 (44%) | 34 (38%) | 35 (45%) | 77 (24%) |  |
| Grade 3 | 98 (41%) | 40 (44%) | 26 (33%) | 212 (67%) |  |
| **Tubule formation** | | | | | |
| 1 | 13 (5%) | 7 (8%) | 4 (5%) | 8 (3%) | 16.883  **0.010** |
| 2 | 91 (38%) | 32 (35%) | 30 (39%) | 87 (27%) |  |
| 3 | 135 (57%) | 51 (57%) | 44 (56%) | 224 (70%) |  |
| **Pleomorphism** | | | | | |
| 1 | 7 (3%) | 2 (2%) | 2 (3%) | 12 (4%) | 44.255  **<0.0001** |
| 2 | 98 (41%) | 36 (40%) | 46 (59%) | 74 (23%) |  |
| 3 | 134 (56%) | 52 (58%) | 30 (38%) | 233 (73%) |  |
| **Mitosis** | | | | | |
| 1 | 97 (41%) | 40 (45%) | 37 (47%) | 67 (21%) | 47.713  **<0.0001** |
| 2 | 53 (22%) | 12 (13%) | 16 (21%) | 64 (20%) |  |
| 3 | 89 (37%) | 38 (42%) | 25 (32%) | 188 (59%) |  |
| **Histologic types** | | | | | |
| No special type (NST) | 129 (54%) | 49 (55%) | 40 (51%) | 244 (76%) | 48.376  **<0.0001** |
| Lobular | 35 (15%) | 12 (13%) | 11 (14%) | 16 (5%) |  |
| Other special types | 13 (5%) | 4 (4%) | 2 (3%) | 3 (1%) |  |
| Mixed NST | 62 (26%) | 25 (28%) | 25 (32%) | 56 (18%) |  |
| **Lymph node status** | | | | | |
| Absent | 146 (61%) | 61 (68%) | 55 (70%) | 184 (58%) | 6.151  0.104 |
| Present | 93 (39%) | 29 (32%) | 23 (30%) | 135 (42%) |  |
| **Lymphovascular invasion** | | | | | |
| Absent | 150 (63%) | 66 (73%) | 53 (68%) | 200 (63%) | 4.243  0.236 |
| Present | 89 (37%) | 24 (27%) | 25 (32%) | 119 (37%) |  |
| **Nottingham Prognostic index** | | | | | |
| Good prognostic group | 74 (31%) | 38 (42%) | 30 (39%) | 62 (19%) | 27.727  **<0.0001** |
| Moderate prognostic group | 131 (55%) | 41 (46%) | 40 (51%) | 194 (61%) |  |
| Poor prognostic group | 34 (14%) | 11 (12%) | 8 (10%) | 63 (20%) |  |
| **ER status** | | | | | |
| Negative | 51 (21%) | 26 (29%) | 10 (13%) | 116 (36%) | 25.556  **<0.0001** |
| Positive | 188 (79%) | 63 (71%) | 68 (87%) | 202 (64%) |  |
| **PR status** | | | | | |
| Negative | 91 (38%) | 31 (36%) | 22 (29%) | 160 (51%) | 18.176  **<0.0001** |
| Positive | 146 (62%) | 54 (64%) | 55 (71%) | 154 (49%) |  |
| **Ki67 expression** | | | | | |
| Low≤14 | 88 (48%) | 33 (45%) | 37 (59%) | 81 (31%) | 21.677  **<0.0001** |
| High>14 | 97 (52%) | 41 (55%) | 26 (41%) | 177 (69%) |  |
| **Molecular subtypes** | | | | | |
| Luminal A | 78 (40%) | 31 (41%) | 32 (49%) | 62 (23%) | 39.444  **<0.0001** |
| Luminal B | 69 (35%) | 20 (27%) | 24 (37%) | 106 (38%) |  |
| Triple negative | 38 (20%) | 17 (23%) | 8 (12%) | 71 (26%) |  |
| HER2 + | 10 (5%) | 7 (9%) | 1 (2%) | 37 (13%) |  |

**Supplementary Table 13.** Clinicopathological significance of RECQL and FOXP3+ (within tumor cell nest, adjacent and distant stroma) co-expression in breast cancers.

|  | **RECQL-/FOXP3-** | **RECQL+/FOXP3+** | **RECQL+/FOXP3-** | **RECQL-/FOXP3+** | **X^2^** |
| --- | --- | --- | --- | --- | --- |
|  |  |  |  |  | ***P*-value** |
| **Tumor size** | | | | | |
| ≤ 2cm | 89 (50%) | 55 (54%) | 35 (54%) | 179 (47%) | 2.145  0.541 |
| > 2cm | 90 (50%) | 47 (46%) | 30 (46%) | 201 (53%) |  |
| **Tumor grade** | | | | | |
| Grade 1 | 28 (16%) | 17 (17%) | 15 (23%) | 38 (10%) | 73.996  **<0.0001** |
| Grade2 | 91 (51%) | 36 (35%) | 33 (51%) | 92 (24%) |  |
| Grade 3 | 60 (33%) | 49 (48%) | 17 (26%) | 250 (66%) |  |
| **Tubule formation** | | | | | |
| 1 | 11 (6%) | 7 (7%) | 4 (7%) | 10 (3%) | 18.933  **0.004** |
| 2 | 72 (40%) | 34 (33%) | 27 (41.5%) | 107 (28%) |  |
| 3 | 96 (54%) | 61 (60%) | 34 (52%) | 263 (69%) |  |
| **Pleomorphism** | | | | | |
| 1 | 6 (3%) | 2 (2%) | 2 (3%) | 13 (3%) | 52.701  **<0.0001** |
| 2 | 84 (47%) | 41 (40%) | 39 (60%) | 90 (24%) |  |
| 3 | 89 (50%) | 59 (58%) | 24 (37%) | 277 (73%) |  |
| **Mitosis** | | | | | |
| 1 | 81 (45%) | 42 (41%) | 34 (52%) | 84 (22%) | 64.897  **<0.0001** |
| 2 | 44 (25%) | 13 (13%) | 15 (23%) | 73 (19%) |  |
| 3 | 54 (30%) | 47 (46%) | 16 (25%) | 223 (59%) |  |
| **Histologic types** | | | | | |
| No special type (NST) | 85 (48%) | 59 (58%) | 30 (46%) | 288 (76%) | 62.008  **<0.0001** |
| Lobular | 31 (17%) | 12 (12%) | 11 (17%) | 20 (5%) |  |
| Other special types | 11 (6%) | 4 (4%) | 2 (3%) | 5 (1%) |  |
| Mixed NST | 52 (29%) | 27 (26%) | 22 (34%) | 67 (18%) |  |
| **Lymph node status** | | | | | |
| Absent | 110 (61%) | 69 (68%) | 47 (72%) | 220 (58%) | 6.915  0.075 |
| Present | 69 (39%) | 33 (32%) | 18 (28%) | 160 (42%) |  |
| **Lymphovascular invasion** | | | | | |
| Absent | 112 (63%) | 71 (70%) | 48 (74%) | 238 (63%) | 4.515  0.211 |
| Present | 67 (37%) | 31 (30%) | 17 (26%) | 142 (37%) |  |
| **Nottingham Prognostic index** | | | | | |
| Good prognostic group | 60 (34%) | 40 (39%) | 27 (41%) | 77 (20%) | 29.807  **<0.0001** |
| Moderate prognostic group | 97 (54%) | 48 (47%) | 33 (51%) | 228 (60%) |  |
| Poor prognostic group | 22 (12%) | 14 (14%) | 5 (8%) | 75 (20%) |  |
| **ER status** | | | | | |
| Negative | 27 (15%) | 28 (28%) | 8 (12%) | 140 (37%) | 37.747  **<0.0001** |
| Positive | 152 (85%) | 73 (72%) | 57 (88%) | 239 (63%) |  |
| **PR status** | | | | | |
| Negative | 54 (31%) | 34 (35%) | 18 (28%) | 198 (53%) | 34.275  **<0.0001** |
| Positive | 123 (69%) | 63 (65%) | 46 (72%) | 177 (47%) |  |
| **Ki67 expression** | | | | | |
| Low≤14 | 72 (52%) | 34 (41%) | 35 (65%) | 98 (32%) | 29.231  **<0.0001** |
| High>14 | 67 (48%) | 48 (59%) | 19 (35%) | 207 (68%) |  |
| **Molecular subtypes** | | | | | |
| Luminal A | 67 (47%) | 32 (38%) | 30 (54%) | 74 (23%) | 53.844  **<0.0001** |
| Luminal B | 54 (37%) | 26 (31%) | 18 (33%) | 121 (37%) |  |
| Triple negative | 19 (13%) | 19 (23%) | 6 (11%) | 90 (27%) |  |
| HER2 + | 5 (3%) | 7 (8%) | 1 (2%) | 42 (13%) |  |

**Supplementary Table 14.** Clinicopathological significance of RECQL and IL17+ TILs (tumor nest) co-expression in breast cancers.

|  | **RECQL-/** IL **17-** | **RECQL-/** IL **17+** | **RECQL+/** IL 17+ | **RECQL+/** IL **17-** | **X^2^** |
| --- | --- | --- | --- | --- | --- |
|  |  |  |  |  | ***P*-value** |
| **Tumor size** | | | | | |
| ≤ 2cm | 241 (47%) | 4 (29%) | 0 (0%) | 74 (51%) | 3.8  0.28 |
| > 2cm | 274 (53%) | 10 (71%) | 1 (100%) | 70 (49%) |  |
| **Tumor grade** | | | | | |
| Grade 1 | 53 (10%) | 1 (7%) | 0 (0%) | 28 (19%) | 14.2  **0.027** |
| Grade2 | 173 (33%) | 4 (29%) | 0 (0%) | 55 (38%) |  |
| Grade 3 | 291 (57%) | 9 (64%) | 1 (100%) | 61 (42%) |  |
| **Tubule formation** | | | | | |
| 1 | 17 (3%) | 0 (0%) | 0 (0%) | 8 (6%) | 4.6  0.59 |
| 2 | 169 (33%) | 4 (28%) | 0 (0%) | 54 (38%) |  |
| 3 | 331 (64%) | 10 (71%) | 1 (100%) | 82 (57%) |  |
| **Pleomorphism** | | | | | |
| 1 | 16 (3%) | 0 (0%) | 0 (0%) | 1 (1%) | 19  **0.003** |
| 2 | 153 (30%) | 6 (43%) | 0 (0%) | 69 (48%) |  |
| 3 | 348 (67%) | 8 (57%) | 1 (100%) | 47 (51%) |  |
| **Mitosis** | | | | | |
| 1 | 144 (28%) | 3 (21%) | 0 (0%) | 64 (44%) | 19.1  **0.004** |
| 2 | 113 (22%) | 1 (7%) | 0 (0%) | 21 (15%) |  |
| 3 | 260 (50%) | 10 (71%) | 1 (100%) | 59 (41%) |  |
| **Histologic types** | | | | | |
| No special type (NST) | 350 (70%) | 11 (79%) | 1(100%) | 77 (54%) | 12.1  0.21 |
| Lobular | 49 (8%) | 1 (7%) | 0 (0%) | 11 (18%) |  |
| Other special types | 13 (1%) | 0 (0%) | 0 (0%) | 5 (3%) |  |
| Mixed NST | 105 (22%) | 2 (14%) | 0 (0%) | 44 (31%) |  |
| **Lymph node status** | | | | | |
| Absent | 306 (59%) | 10 (71%) | 0 (0%) | 97 (67%) | 5.3  0.15 |
| Present | 211 (41%) | 4 (29%) | 1 (100%) | 47 (33%) |  |
| **Lymphovascular invasion** | | | | | |
| Absent | 325 (63%) | 8 (57%) | 0 (0%) | 97 (67%) | 3.0  0.39 |
| Present | 192 (37%) | 4 (43%) | 1 (100%) | 47 (33%) |  |
| **Nottingham Prognostic index** | | | | | |
| Good prognostic group | 125 (24%) | 2 (14%) | 0 (0%) | 55 (38%) | 19.7  **0.003** |
| Moderate prognostic group | 296 (57%) | 11 (79%) | 0 (0%) | 71 (49%) |  |
| Poor prognostic group | 96 (19%) | 1 (7%) | 1 (100%) | 18 (13%) |  |
| **ER status** | | | | | |
| Negative | 152 (29%) | 7 (50%) | 0 (0%) | 46 (32%) | 10.2  **0.01** |
| Positive | 364 (71%) | 7 (50%) | 1 (100%) | 96 (68%) |  |
| **PR status** | | | | | |
| Negative | 235 (46%) | 8 (57%) | 0(0%) | 32 (36%) | 20.1  **<0.0001** |
| Positive | 276 (54%) | 6 (43%) | 1 (100%) | 57 (64%) |  |
| **HER2** |  |  |  |  |  |
| Negative | 430 (84%) | 12 (86%) | 1 (100%) | 129 (90%) | 3.4  0.32 |
| Positive | 81 (16%) | 2 (14%) | 0 (0%) | 14 (10%) |  |
| **Ki67 expression** | | | | | |
| Low≤14 | 146 (35%) | 4 (40%) | 0 (0%) | 61 (51%) | 10.3  **0.016** |
| High>14 | 266 (65%) | 6(60%) | 1 (100%) | 58 (49%) |  |
| **Molecular subtypes** | | | | | |
| Luminal A | 119 (27%) | 3 (25%) | 0 (0%) | 55 (46%) | 22.8  **0.007** |
| Luminal B | 43 (10%) | 1 (8%) | 0 (0%) | 7 (6%) |  |
| Triple negative | 98 (22%) | 6 (50%) | 0 (0%) | 22 (18%) |  |
| HER2 + | 178 (41%) | 2 (17%) | 1 (100%) | 37 (31%) |  |

**Supplementary Table 15.** Clinicopathological significance of RECQL and IL17+ TILs (adjacent stroma) co-expression in breast cancers.

|  | **RECQL-/** IL **17-** | **RECQL-/** IL **17+** | **RECQL+/** IL 17+ | **RECQL+/** IL **17-** | **X^2^** |
| --- | --- | --- | --- | --- | --- |
|  |  |  |  |  | ***P*-value** |
| **Tumor size** | | | | | |
| ≤ 2cm | 88 (41%) | 162 (50%) | 45 (59%) | 26 (43%) | 9.2  0.026 |
| > 2cm | 127 (59%) | 162 (50%) | 31 (41%) | 70 (57%) |  |
| **Tumor grade** | | | | | |
| Grade 1 | 13 (6%) | 41 (13%) | 20 (26%) | 8 (13%) | 25.2  **<0.0001** |
| Grade2 | 70 (33%) | 113 (35%) | 25 (33%) | 24 (39%) |  |
| Grade 3 | 132 (61%) | 170 (52%) | 31 (41%) | 29 (48%) |  |
| **Tubule formation** | | | | | |
| 1 | 4 (2%) | 13 (4%) | 4 (5%) | 4 (7%) | 5.1  0.53 |
| 2 | 71 (33%) | 107 (33%) | 29 (38%) | 20 (33%) |  |
| 3 | 140 (65%) | 204 (63%) | 43 (57%) | 37 (61%) |  |
| **Pleomorphism** | | | | | |
| 1 | 3 (3%) | 13 (4%) | 1 (1%) | 0 (0%) | 24.9  **<0.0001** |
| 2 | 58 (27%) | 106 (33%) | 41 (54%) | 23 (38%) |  |
| 3 | 154 (72%) | 205 (63%) | 34 (45%) | 38 (62%) |  |
| **Mitosis** | | | | | |
| 1 | 42 (20%) | 108 (33%) | 37 (49%) | 24 (39%) | 28.7  **<0.0001** |
| 2 | 47 (22%) | 69 (21%) | 10 (13%) | 9 (15%) |  |
| 3 | 126 (58%) | 147 (45%) | 29 (38%) | 28 (46%) |  |
| **Histologic types** | | | | | |
| No special type (NST) | 151 (70%) | 213 (66%) | 39 (51%) | 36 (59%) | 15.5  0.076 |
| Lobular | 17 (8%) | 34 (10%) | 11 (14%) | 6 (10%) |  |
| Other special types | 1 (1%) | 12 (4%) | 3 (4%) | 2 (3%) |  |
| Mixed NST | 46 (21%) | 65 (20%) | 23 (30%) | 17 (28%) |  |
| **Lymph node status** | | | | | |
| Absent | 129 (60%) | 193 (60%) | 49 (65%) | 42 (69%) | 2.3  0.5 |
| Present | 86 (40%) | 131 (40%) | 27 (35%) | 19 (31%) |  |
| **Lymphovascular invasion** | | | | | |
| Absent | 145 (67%) | 195 (60%) | 53 (70%) | 37 (60%) | 4.4  0.21 |
| Present | 70 (33%) | 129 (40%) | 23 (30%) | 24 (39%) |  |
| **Nottingham Prognostic index** | | | | | |
| Good prognostic group | 49 (23%) | 82 (25%) | 31 (41%) | 20 (33%) | 13.1  **0.042** |
| Moderate prognostic group | 121 (56%) | 189 (58%) | 37 (49%) | 31 (51%) |  |
| Poor prognostic group | 45 (21%) | 53 (16%) | 8 (11%) | 10 (16%) |  |
| **ER status** | | | | | |
| Negative | 81 (38%) | 79 (24%) | 11 (14%) | 20 (33%) | 19.8  **<0.0001** |
| Positive | 133 (62%) | 245 (76%) | 65 (86%) | 41 (67%) |  |
| **PR status** | | | | | |
| Negative | 116 (54%) | 130 (41%) | 21 (28%) | 22 (37%) | **19.4**  **<0.0001** |
| Positive | 98 (56%) | 189 (59%) | 54 (72%) | 38 (63%) |  |
| **HER2** |  |  |  |  |  |
| Negative | 181 (85%) | 269 (84%) | 68 (91%) | 54 (89%) | 2.8  0.42 |
| Positive | 31 (15%) | 52 (16%) | 0 (9%) | 7 (11%) |  |
| **Ki67 expression** | | | | | |
| Low≤14 | 49 (28%) | 101 (40%) | 34 (58%) | 27 (47%) | 18.4  **<0.0001** |
| High>14 | 124 (72%) | 151 (60%) | 25 (42%) | 31 (53%) |  |
| **Molecular subtypes** | | | | | |
| Luminal A | 39 (21%) | 83 (31%) | 32 (52%) | 23 (41%) | 34.7  **<0.0001** |
| Luminal B | 70 (38%) | 113 (42%) | 19 (31%) | 16 (29%) |  |
| Triple negative | 59 (31%) | 46 (17%) | 8 (13%) | 13 (23%) |  |
| HER2 + | 18 (10%) | 26 (10%) | 3 (4%) | 4 (7%) |  |

**Supplementary Table 16.** Clinicopathological significance of RECQL and IL17+ (tumor nest + adjacent stroma) co-expression in breast cancers.

|  | **RECQL-/** IL **17-** | **RECQL-/** IL **17+** | **RECQL+/** IL 17+ | **RECQL+/** IL **17-** | **X^2^** |
| --- | --- | --- | --- | --- | --- |
|  |  |  |  |  | ***P*-value** |
| **Tumor size** | | | | | |
| ≤ 2cm | 118 (43%) | 129 (50%) | 31 (57%) | 43 (47%) | 5.1  0.16 |
| > 2cm | 156 (57%) | 128 (50%) | 23 (43%) | 48 (53%) |  |
| **Tumor grade** | | | | | |
| Grade 1 | 19 (7%) | 35 (14%) | 11 (20%) | 17 (19%) | 19.6  **0.003** |
| Grade2 | 89 (32%) | 88 (34%) | 19 (35%) | 36 (40%) |  |
| Grade 3 | 166 (61%) | 134 (52%) | 24 (45%) | 38 (42%) |  |
| **Tubule formation** | | | | | |
| 1 | 6 (2%) | 11 (4%) | 2 (4%) | 6 (7%) | 6.01  0.42 |
| 2 | 89 (35%) | 84 (33%) | 19 (35%) | 35 (39%) |  |
| 3 | 179 (65%) | 162 (63%) | 33 (61%) | 50 (55%) |  |
| **Pleomorphism** | | | | | |
| 1 | 3 (1%) | 13 (5%) | 0 (0%) | 1 (1%) | 27.9  **<0.0001** |
| 2 | 76 (28%) | 83 (32%) | 26 (48%) | 43 (47%) |  |
| 3 | 195 (71%) | 161 (63%) | 28 (52%) | 47 (52%) |  |
| **Mitosis** | | | | | |
| 1 | 59 (22%) | 88 (34%) | 23 (43%) | 41 (45%) | 27.2  **<0.0001** |
| 2 | 57 (21%) | 57 (22%) | 8 (15%) | 13 (14%) |  |
| 3 | 158 (58%) | 112 (44%) | 23 (43%) | 37 (41%) |  |
| **Histologic types** | | | | | |
| No special type (NST) | 192 (70%) | 169 (66%) | 31 (57%) | 47 (52%) | 20.12  **0.017** |
| Lobular | 21 (8%) | 29 (11%) | 7 (13%) | 11 (12%) |  |
| Other special types | 2 (1%) | 11 (4%) | 2 (4%) | 3 (3%) |  |
| Mixed NST | 59 (22%) | 48 (19%) | 14 (26%) | 30 (33%) |  |
| **Lymph node status** | | | | | |
| Absent | 170 (62%) | 146 (57%) | 34 (63%) | 63 (69%) | 4.7  0.19 |
| Present | 104 (38%) | 111 (43%) | 20 (37%) | 28 (31%) |  |
| **Lymphovascular invasion** | | | | | |
| Absent | 182 (66%) | 151 (59%) | 34 (63%) | 63 (69%) | 4.8  0.187 |
| Present | 92 (34%) | 106 (41%) | 20 (37%) | 28 (31%) |  |
| **Nottingham Prognostic index** | | | | | |
| Good prognostic group | 63 (30%) | 64 (25%) | 22 (41%) | 33(36%) | 13.7  **0.03** |
| Moderate prognostic group | 158 (58%) | 149 (58%) | 23 (43%) | 48 (53%) |  |
| Poor prognostic group | 53 (19%) | 44 (17%) | 9 (17%) | 10 (11%) |  |
| **ER status** | | | | | |
| Negative | 106 (39%) | 53 (21%) | 8 (15%) | 24 (26%) | 27.3  **<0.0001** |
| Positive | 167 (61%) | 204 (79%) | 46 (85%) | 67 (74%) |  |
| **PR status** | | | | | |
| Negative | 142 (53%) | 101 (40%) | 14 (26%) | 32 (36%) | 20.1  **<0.0001** |
| Positive | 127 (47%) | 204 (60%) | 40 (74%) | 57 (64%) |  |
| **HER2** |  |  |  |  |  |
| Negative | 225 (84%) | 217 (85%) | 47 (89%) | 83 (91%) | 3.6  0.329 |
| Positive | 44 (16%) | 39 (15%) | 6 (11%) | 8 (8%) |  |
| **Ki67 expression** | | | | | |
| Low≤14 | 64 (29%) | 86 (23%) | 22 (52%) | 39 (50%) | **17.3**  **0.001** |
| High>14 | 156 (71%) | 116 (57%) | 20 (48%) | 39 (50%) |  |
| **Molecular subtypes** | | | | | |
| Luminal A | 49 (21%) | 37 (34%) | 20 (44%) | 35 (46%) | 36.8  **<0.0001** |
| Luminal B | 26 (11%) | 18 (8%) | 2 (4%) | 5 (7%) |  |
| Triple negative | 72 (31%) | 32 (15%) | 6 (13%) | 16 (21%) |  |
| HER2 + | 89 (38%) | 91 (41%) | 17 (38%) | 21 (27%) |  |

**Supplementary Table 17.** Clinicopathological significance of RECQL and PDL1+ (TILs) co-expression in breast cancers.

|  | **RECQL-/PD1-** | **RECQL+/PD1+** | **RECQL+/PD1-** | **RECQL-/PDL1+** | **X^2^** |
| --- | --- | --- | --- | --- | --- |
|  |  |  |  |  | ***P*-value** |
| **Tumor size** | | | | | |
| ≤ 2cm | 104 (50%) | 31 (46%) | 44 (63%) | 159 (46%) | 7.22  0.065 |
| > 2cm | 103 (50%) | 37 (54%) | 26 (37%) | 188 (54%) |  |
| **Tumor grade** | | | | | |
| Grade 1 | 41 (20%) | 5 (7%) | 16 (23%) | 26 (7%) | 59.089  **<0.0001** |
| Grade2 | 85 (41%) | 26 (38%) | 34 (49%) | 96 (28%) |  |
| Grade 3 | 81 (39%) | 37 (55%) | 20 (28%) | 225 (65%) |  |
| **Tubule formation** | | | | | |
| 1 | 19 (9%) | 2 (3%) | 7 (10%) | 5 (1%) | 34.291  **<0.0001** |
| 2 | 79 (38%) | 24 (35%) | 26 (37%) | 96 (28%) |  |
| 3 | 109 (53%) | 42 (62%) | 37 (53%) | 246 (71%) |  |
| **Pleomorphism** | | | | | |
| 1 | 10 (5%) | 2 (3%) | 1 (1%) | 6 (2%) | 89.639  **<0.0001** |
| 2 | 97 (47%) | 16 (23%) | 49 (70%) | 76 (22%) |  |
| 3 | 100 (48%) | 50 (74%) | 20 (29%) | 265 (76%) |  |
| **Mitosis** | | | | | |
| 1 | 82 (40%) | 23 (34%) | 35 (50%) | 80 (23%) | 48.374  **<0.0001** |
| 2 | 53 (25%) | 10 (15%) | 17 (24%) | 63 (18%) |  |
| 3 | 72 (35%) | 35 (51%) | 18 (26%) | 204 (59%) |  |
| **Histologic types** | | | | | |
| No special type (NST) | 110 (53%) | 44 (65%) | 30 (43%) | 270 (78%) | 60.986  **<0.0001** |
| Lobular | 24 (12%) | 8 (12%) | 10 (14%) | 21 (6%) |  |
| Other special types | 11 (5%) | 2 (3%) | 7 (10%) | 3 (1%) |  |
| Mixed NST | 62 (30%) | 14 (20%) | 23 (33%) | 53 (15%) |  |
| **Lymph node status** | | | | | |
| Absent | 124 (60%) | 42 (62%) | 54 (77%) | 198 (57%) | 9.897  **0.019** |
| Present | 83 (40%) | 26 (38%) | 16 (23%) | 149 (43%) |  |
| **Lymphovascular invasion** | | | | | |
| Absent | 138 (67%) | 41 (60%) | 55 (79%) | 210 (60%) | 9.331  **0.025** |
| Present | 69 (33%) | 27 (40%) | 15 (21%) | 137 (40%) |  |
| **Nottingham Prognostic index** | | | | | |
| Good prognostic group | 65 (31%) | 18 (26%) | 35 (50%) | 66 (19%) | 38.292  **<0.0001** |
| Moderate prognostic group | 117 (57%) | 40 (59%) | 31 (44%) | 208 (60%) |  |
| Poor prognostic group | 25 (12%) | 10 (15%) | 4 (6%) | 73 (21%) |  |
| **ER status** | | | | | |
| Negative | 38 (18%) | 24 (36%) | 6 (9%) | 135 (39%) | 43.105  **<0.0001** |
| Positive | 169 (82%) | 43 (64%) | 63 (91%) | 211 (61%) |  |
| **PR status** | | | | | |
| Negative | 78 (38%) | 26 (40%) | 20 (29%) | 188 (55%) | 25.655  **<0.0001** |
| Positive | 127 (62%) | 39 (60%) | 49 (71%) | 153 (45%) |  |
| **Ki67 expression** | | | | | |
| Low≤14 | 80 (47%) | 21 (37%) | 33 (61%) | 81 (30%) | 25.596  **<0.0001** |
| High>14 | 90 (53%) | 36 (63%) | 21 (39%) | 191 (70%) |  |
| **Molecular subtypes** | | | | | |
| Luminal A | 73 (41%) | 18 (31%) | 29 (52%) | 60 (21%) | 53.87  **<0.0001** |
| Luminal B | 70 (39%) | 17 (29%) | 21 (37%) | 108 (37%) |  |
| Triple negative | 25 (14%) | 19 (33%) | 5 (9%) | 89 (30%) |  |
| HER2 + | 11 (6%) | 4 (7%) | 1 (2%) | 36 (12%) |  |

**Supplementary Table 18.** Correlation between REQL1 and immune markers.

| **Variables** | **REQL1 expression** | | **X^2^**  ***P*-value** |
| --- | --- | --- | --- |
|  | **Low** | **High** |  |
| **PDL1 positivity**  Negative  Positive | 90 (18%)  41 (82%) | 15 (12%)  111 (88%) | 2.4  0.08 |
| **PD1 positivity**  Negative  Positive | 207 (32%)  347 (63%) | 66 (39%)  68 (49%) | 8.2  **0.004** |
| **Foxp3**  Negative  Positive | 179 (37%)  379 (68%) | 70 (51%)  102 (61%) | 3.0  0.08 |
| **CD8**  Negative  Positive | 66 (12%)  471 (88%) | 23 (15%)  129 (85%) | 0.8  0.35 |
| **IL17**  Negative  Positive | 274 (52%)  257 (48%) | 91 (63%)  54 (37%) | 5.7  **0.01** |

**Supplementary Table 19.** Multivariate analysis REQL1/PDL1 co-expression in breast cancer

| **Parameters** | **BCSS** | | |
| --- | --- | --- | --- |
|  | Hazard ratio | 95% (CI) | *P-value* |
| **RECQL** | 0.49 | 0.28-0.89 | ***0.018*** |
| **CD8** | 0.99 | 0.99-1.0 | ***0.049*** |
| **FOXP3** | 1.8 | 1.77-2.9 | ***0.007*** |
| **PDL1** | 0.82 | 0.5-1.3 | *0.41* |
| **IL17** | 1.1 | 0.83-1.7 | *0.3* |
| **Tumor size** | 2.5 | 1.8-2.9 | ***<0.0001*** |
| **Lymph node status** | 1.8 | 1.2-2.6 | ***0.001*** |

BCSS, breast cancer specific cancer. 95%CI, 95% confidence interval. Significant p values are in **bold.**

**Supplementary Table 21.** Whole genome and whole exome sequencing in patients with RECQL germ-line mutations.

| P# | Whole Genome Sequencing Mean Coverage | Whole Exome Sequencing Mean Coverage | Coding Region Size (Mbp) | TMB based on Exome Sequencing | Microsatellites 60x< | Unstable Microsatellites 60x< | Proportion of Unstable Microsatellites 60x< | LOH_  Score | TAI_  Score | LST_  Score | HRD_  Score | Comment |
| --- | --- | --- | --- | --- | --- | --- | --- | --- | --- | --- | --- | --- |
| 1 | 8x | 126x | 34 | 8.1 | 1255 | 355 | 0.28 | 0 | 2 | 3 | 5 |  |
| 2 | 15x | 199x | 34 | 10.6 | 493 | 90 | 0.18 | 0 | 6 | 9 | 15 |  |
| 3 | 11x | 21x | 34 | 5.4 | 540 | 150 | 0.28 | 19 | 19 | 21 | 59 | CDK12 somatic mutation, NM_016507.4:c.3199C>T, NP_057591.2:p.Arg1067Ter |
| 4 | 24x | 223x | 34 | 10.9 | 388 | 78 | 0.20 | 0 | 11 | 22 | 33 |  |
| 5 | 35x | 252x | 34 | 15.2 | 859 | 146 | 0.17 | 0 | 11 | 12 | 23 |  |
